# Advances in Electrical Materials for Bone and Cartilage Regeneration: Developments, Challenges, and Perspectives

**DOI:** 10.1002/advs.202411209

**Published:** 2025-02-14

**Authors:** Yubin Yao, Xi Cui, Shenglong Ding, Ketao Wang, Mingzhu Zhang

**Affiliations:** ^1^ Department of Foot and Ankle Surgery Beijing Tongren Hospital Capital Medical University Beijing 100730 China

**Keywords:** electrical biomaterials, electrical stimulation, endogenous electric fields, nanogenerators, piezoelectric materials

## Abstract

Severe bone and cartilage defects caused by trauma are challenging to treat, often resulting in poor outcomes. An endogenous electric field (EnEF) is crucial for bone regeneration, making electrical materials a promising therapy. This review provides a comprehensive overview of the role of bioelectric signals in bone and cartilage cells, alongside recent advancements in electrical biomaterials, with particular emphasis on nanogenerators, piezoelectric materials, triboelectric scaffolds, and zwitterionic hydrogels. It further investigates the impact of these electrical biomaterials on bone and cartilage regeneration, as well as the applications of both endogenous and exogenous electrical stimulation (ES) and the mechanisms underlying ES‐induced cellular and molecular responses. Finally, the review underscores future directions for ES systems in tissue engineering, emphasizing the critical importance of integrating structural integrity, mechanical properties, and electrical signal delivery into intelligent implantable scaffolds.

## Graphical abstract

This review explores the challenges of treating bone and cartilage defects, emphasizing the role of endogenous electric fields in bone and cartilage regeneration. It highlights recent advancements in electroactive biomaterials, including nanogenerators, piezoelectric materials, triboelectric scaffold, and zwitterionic hydrogels. Key applications of electrical stimulation and self‐powered systems are also explored, as future directions for intelligent, implantable scaffolds in tissue engineering.

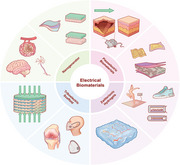

## 1 Introduction

Bone and cartilage injuries caused by factors such as trauma, tumors, strain, and osteoarthritis are becoming increasingly common in daily life. Among the various treatment methods, such as bone transplantation, membrane‐induced regeneration, gene therapy, and tissue engineering, bone tissue regeneration has received widespread attention from researchers owing to its low surgical difficulty and significant repair effects.^[^ ^1^ ^]^ The key to bone tissue engineering lies in regulating the directional differentiation of stem cells to promote bone regeneration. However, the development of ideal bone tissue regeneration materials still faces enormous challenges. Recently, the use of implanted synthetic scaffolds to mimic the osteogenic microenvironment has emerged as a promising approach for modulating the extracellular environment at wound sites to enhance bone regeneration.^[^ ^2^ ^]^ However, because of the limited effectiveness of bone and cartilage repair after implantation, relying exclusively on biological materials for in vivo restoration is insufficient. One contributing factor is that the critical influence of physical signals such as bioelectricity within the bone and cartilage microenvironments is frequently neglected.^[^ ^3^ ^]^ Lacking endogenous electric fields (EnEFs) and the ability to detect mechanical stimuli, these tissues suffer further microenvironmental damage.^[^ ^4^ ^]^ Fortunately, mechanical stimulation can be transformed into electrical signals by modifying the activity of the mechanically sensitive channels associated with piezoelectric materials.^[^ ^5^ ^]^

Electrical stimulation (ES) has garnered significant attention as a crucial biophysical regulatory factor and non‐pharmacological intervention in clinical settings, owing to its remarkable capacity to influence cell activity and promote tissue repair.^[^ ^6^, ^7^, ^8^ ^]^ Emerging nanogenerators, which are considered promising candidates for future energy technologies, can convert mechanical energy into electrical energy via frictional or piezoelectric effects.^[^ ^9^ ^]^ Piezoelectric nano‐materials are functional substances that show great potential for nano‐scale conversion of mechanical energy into electrical signals. Furthermore, researchers have designed friction electric scaffolds (TESs) that facilitate the development of dense and mature cartilage by inoculating chondrocytes, followed by subcutaneous implantation.^[^ ^10^ ^]^ Electrical nano‐materials have extensive applications in energy‐harvesting, sensors, actuators, resonators, and medical detection devices owing to their exceptional electromechanical properties, catalytic performance, and sensitivity to stimuli.^[^ ^11^ ^]^ Micro/nanoelectrical materials demonstrate distinctive electrical and chemical behaviors within the realm of biomedical engineering, especially in bone and cartilage injury treatments. Importantly, osteochondral defects implanted with micro/nanoelectrical materials in vivo show good hyaluronic acid cartilage regeneration and complete cartilage healing.^[^ ^5^, ^12^ ^]^

Although electrically active materials have shown excellent results in bone and cartilage repair, particularly in bone, many studies have highlighted their limitations. Traditional ES therapy requires bulky equipment, reducing patient comfort and limiting personalized treatment, which hinders its clinical use.^[^ ^13^ ^]^ Additionally, traditional energy‐storage devices are rigid and difficult to integrate into wearable technologies. To address this, flexible electrical materials capable of converting mechanical energy, such as body movement, into electricity have gained popularity due to their adaptability, simplicity, and lightweight nature.^[^ ^14^ ^]^ Research on flexible micro‐/nanoelectronic devices has focused primarily on improving electrical performance or exploring various manufacturing methods such as melt spinning, solution spinning, electrospinning, and 3D printing.^[^ ^15^, ^16^ ^]^ Structural designs, including microstructures, core–shell structures, and porous structures, have also been explored to enhance electrical output.^[^ ^17^ ^]^ However, there are still some key issues associated with electrical micro/nano‐materials, such as low electric output performance, poor washing resistance, and poor durability.

Electrical biomaterials are essential for the regeneration of bone and cartilage, as they replicate endogenous electrical signals.^[^ ^18^ ^]^ These materials effectively stimulate cellular activities, facilitate cell proliferation and directed differentiation, regulate the synthesis of extracellular matrix components, promote cell migration, and expedite tissue repair processes.^[^ ^19^ ^]^ Additionally, electrical biomaterials can reduce inflammatory responses, thus establishing an optimal microenvironment conducive to regeneration. They demonstrate excellent biocompatibility and controllable directional characteristics, enabling them to fulfill diverse regenerative requirements.^[^ ^20^, ^21^ ^]^ These electrically active materials must be biocompatible, offering minimal cytotoxicity while supporting cell adhesion and growth. Additionally, they require sufficient mechanical strength to withstand physiological loads and should possess tailored electrical properties to optimize cellular responses and promote successful integration. However, several key bottlenecks hinder progress in this field. There are challenges in selecting and fabricating materials that balance conductivity, mechanical properties, and biocompatibility. Furthermore, regulatory hurdles delay the clinical application of new biomaterials, and ensuring the long‐term stability and performance of these materials within the biological environment remains a significant concern. Addressing these needs and overcoming these challenges is crucial for advancing the use of electrically active materials in clinical applications for bone and cartilage repair.

This article systematically reviews the working principles, device design mechanics, classifications, biological effects, mechanisms, and applications of piezoelectric materials, nanogenerators, zwitterionic hydrogels, and triboelectric scaffolds for bone and cartilage tissue engineering. The upcoming sections discuss the prospects of electric materials and their potential roles as foundational elements that can inspire and influence advancements in bone and cartilage treatment applications.

## 2 Emerging Electrical Biomaterials

### 2.1 Overview of Electrical Biomaterials

With improvements in living standards and technological advances, there is a growing demand for convenient and comfortable disease diagnosis and health monitoring. Traditional medical electronics often fall short of modern demands due to their large size and limited power supply. For instance, although Dr. Paul Zoll's 1952 invention of the first human heart pacemaker excited both the medical community and broader society, its substantial dimensions hindered further progress. Ongoing enhancements have rendered contemporary heart pacemakers suitable for internal implantation. However, their operational lifespan remains restricted by their battery capacity. Analogous challenges persist across diverse facets of healthcare, such as blood sugar or blood pressure monitoring, which depend on specialized medical apparatus.

**Figure 1:**
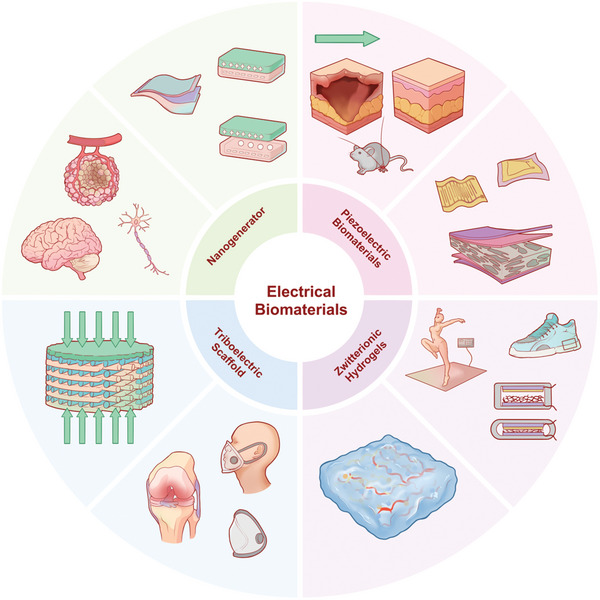
Principles and applications of electrical biomaterials. Electrical biomaterials can be divided into four main categories: nanogenerators, piezoelectric biomaterials, triboelectric scaffolds, and zwitterionic hydrogels. 1. Nanogenerators: Nanogenerators convert small mechanical movements into electrical energy, leveraging the piezoelectric or triboelectric effect. In neurological applications, nanogenerators could potentially harvest bio‐mechanical energy to power implants or wearable devices, which can monitor brain activity, stimulate nerve cells, or facilitate nerve repair. These could also play a role in pacemakers or other implantable devices that harvest energy from body movements. 2. Piezoelectric Biomaterials: Piezoelectric materials generate an electric charge in response to applied mechanical stress. In biomedical applications, piezoelectric biomaterials are useful for wound healing, tissue engineering, and regenerative medicine, where mechanical stimuli are converted into electrical signals to encourage cellular response. 3. Triboelectric Scaffolds: Triboelectric scaffolds leverage the triboelectric effect, where two materials generate charge when rubbed together. These scaffolds are especially promising for bone and cartilage regeneration, as mechanical movements during joint activity can generate electric stimulation, which promotes cell growth and tissue repair. 4. Zwitterionic Hydrogels: Zwitterionic hydrogels are hydrogels that carry both positive and negative charges. They are highly flexible, biocompatible, and resistant to protein fouling, making them ideal for wearable devices or flexible electronics. They are commonly used in bioelectronics and soft robotics, as they can maintain function while enduring repetitive motion. The hydrogel's conductive properties enable it to act as a sensor or actuator in applications like wearable sports equipment or medical monitoring devices. Each of these categories represents a unique way in which mechanical energy or environmental stimuli are converted into electrical energy to influence biological processes or power devices. The concept of electrical biomaterials holds promise in applications ranging from neural interfaces to regenerative medicine and wearable health devices.

The emergence and evolution of electroactive biomaterials offer an advantageous foundation for advancing miniaturization efforts alongside self‐sustaining systems in associated electronic devices. This section provides a comprehensive review encompassing composition analysis, structural examination, electrical property assessment, and application exploration pertaining to various electrically active biomaterials within the biomedical domain (**Figure** **1**).

### 2.2 Nanogenerators

As medical technology advances, the need for more precise, miniaturized therapeutic devices grows. However, most still rely on external power sources, creating significant challenges during treatment.^[^ ^22^ ^]^ To address this limitation, nanogenerators have been developed. In the absence of an external power supply, nanogenerators can generate self‐sustaining power by converting various forms of mechanical energy into electrical energy.^[^ ^23^, ^24^ ^]^ Based on this operational principle, nanogenerators can be classified into piezoelectric nanogenerators (PENGs) and triboelectric nanogenerators (TENGs).^[^ ^25^, ^26^ ^]^

**Figure 2:**
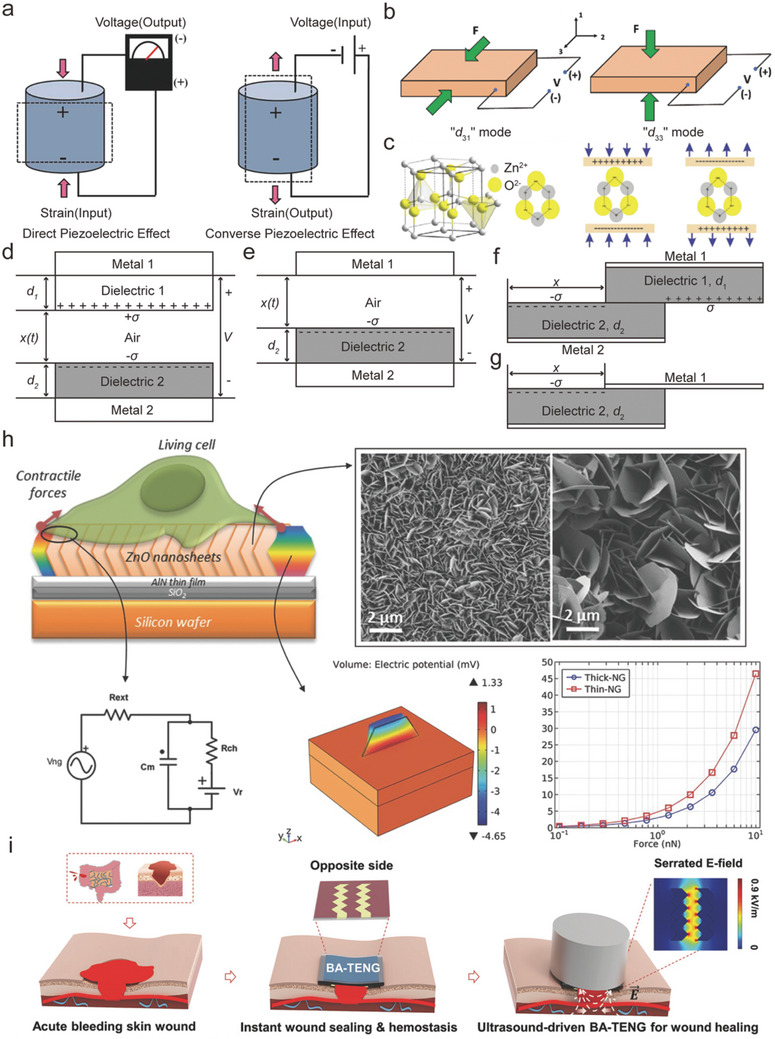
a) Direct and converse piezoelectric effect. Reproduced with permission.^[^ ^30^ ^]^ Copyright 2018, Wiley‐VCH. b) “*d*_31_” mode means that the force direction is perpendicular to poling voltage direction. In contrast, the force direction is parallel to poling voltage direction in “*d*_33_” mode. Reproduced with permission.^[^ ^29^ ^]^ Copyright 2021, Wiley‐VCH. c) ZnO fiber‐zinc ore structural model, piezoelectric potential in compression and tensile mode. Reproduced with permission.^[^ ^32^ ^]^ Copyright 2022, Wiley‐VCH. Theoretical models for d) dielectric‐to‐dielectric contact‐mode TENG and e) conductor‐to‐dielectric contact‐mode TENG.^[^ ^49^ ^]^ Theoretical models for f) dielectric‐to‐dielectric sliding‐mode TENG and g) conductor‐to‐dielectric sliding‐mode TENG. Reproduced with permission.^[^ ^48^ ^]^ Copyright 2013, Wiley‐VCH. h) Piezoelectric nanogenerator (PENG), based on ZnO nanosheets (NSs), interacts with living cells to induce a local electric field that self‐stimulates and modulates cell activity. Reproduced with permission.^[^ ^53^ ^]^ Copyright 2017, Wiley‐VCH. i) A bioadhesive triboelectric nanogenerator (BA‐TENG) for instant and robust wound sealing and ultrasound‐driven accelerated wound healing. Reproduced with permission.^[^ ^54^ ^]^ Copyright 2023, Wiley‐VCH.

PENGs consist of piezoelectric materials, flexible substrates, and connecting electrodes.^[^ ^27^, ^28^ ^]^ Piezoelectric materials are typically crystals with noncentrosymmetric structures, indicating that their lattices lack inversion symmetry, which is a necessary condition for the piezoelectric effect.^[^ ^29^ ^]^ When mechanical stress is applied to a piezoelectric material, it induces deformation of the internal lattice, and the charge centers of the anions and cations separate, thereby generating an electric dipole moment on the material and further generating an internal potential. The charge produced is directly related to the applied mechanical stress; this phenomenon is known as the direct piezoelectric effect. In contrast, when an electric field is applied to a piezoelectric material, it induces mechanical deformation, which is known as the converse piezoelectric effect^[^ ^29^, ^30^ ^]^ (**Figure** **2**a,b). Using ZnO crystals as an illustration (Figure 2c), the arrangement of tetrahedrally coordinated Zn^2+^ and O_2_^−^ occurs in layers along the axis, with the charge centers of both anions and cations aligning without any stress influence. When an external force is applied to one corner of the tetrahedron, the displacement between the negative and positive charge centers creates an electric dipole. Furthermore, the cumulative effect of the dipole moments from all the units within the crystal results in a piezoelectric field that induces a potential difference in line with the direction of the crystal strain, which is referred to as the piezoelectric potential. Once the crystal is linked to an external load, the electrons within the circuit are compelled to move, partially mitigating the piezoelectric potential.^[^ ^31^, ^32^ ^]^ Consequently, when a dynamic external force periodically modifies the piezoelectric potential, continuous current pulses are generated through the external circuit. In practical applications, the chemical vapor deposition (CVD) method is a common approach to achieve mass production of PENGs, but inevitable defects occur during the production process, which leads to the piezoelectric shielding effect and significantly reduces product performance.^[^ ^33^ ^]^ In this regard, in addition to defect passivation for control,^[^ ^34^ ^]^ piezoelectric performance can be enhanced by embedding an organic molecular layer with large mechanical flexibility and structural asymmetry as the active layer in the material.^[^ ^35^ ^]^ Over recent decades, PENGs have evolved from ZnO nanowires, nanocomposites such as BaTiO_3_ to materials such as polyvinylidene fluoride (PVDF), graphene, and lead‐free NaKNbO_3_.^[^ ^36^, ^37^, ^38^, ^39^, ^40^, ^41^, ^42^ ^]^ The update and improvement of materials not only increases the output of the PENG, but also significantly enhances the plasticity, safety, and miniaturization of the product. These performance upgrades further expand the application scope of PENGs in the medical and health fields, particularly for miniaturized electronic devices.^[^ ^12^, ^43^ ^]^

The fundamental operating principle of TENGs is founded upon the coupling of contact electrification and electrostatic induction.^[^ ^44^, ^45^ ^]^ When two materials with disparate electron affinities come into contact and subsequently separate, one has a propensity to acquire electrons (becoming negatively charged), whereas the other tends to lose electrons (becoming positively charged). This phenomenon is called contact electrification or the triboelectric effect. Since their initial inception in 2012,^[^ ^46^ ^]^ based on the classification of motion direction and capacitance variation, four fundamental working modalities of TENGs have been proposed and can be categorized into two types: contact separation (Figure 2d,e) and sliding (Figure 2f,g).^[^ ^47^, ^48^ ^]^ The potential difference between the two materials was determined using an external circuit for electrical energy‐harvesting. When the materials are in contact and then separated repeatedly (through mechanical movements such as tapping or rubbing), the processes of contact electrification and electrostatic induction recur, thereby generating an alternating current.^[^ ^49^ ^]^ The electrical output produced by the TENG exhibits a high voltage and low current; however, its triboelectric characteristics are readily influenced by the surface properties of the materials. This issue can be tackled through surface modification approaches, such as injecting charged particles and controlling defects, or it can be mitigated by physical methods, such as material surface plasma treatment. Additionally, the output performance of a TENG can be enhanced by augmenting the friction area, for instance, by modifying the simple graphene structure into a wrinkled graphene structure. TENGs can be fabricated on flexible substrates with curved or irregular surfaces. This high flexibility is beneficial for applications that require conformal energy collection or integration into wearable or flexible electronic devices.^[^ ^46^ ^]^

The application of nanogenerators has extensive applications. In the medical field, nanogenerators tailored for diverse application scenarios and diagnostic and therapeutic requirements have demonstrated remarkable performance in treating neuromotor system disorders, expediting skin wound healing, and monitoring physiological indicators.^[^ ^50^, ^51^, ^52^ ^]^ Gonzalo et al. developed a PENG based on 2D ZnO nanosheets (Figure 2h). This nanogenerator can interact with living cells to form a local electric field. Moreover, it can self‐stimulate and regulate cell activities without the assistance of any external chemical or physical stimuli. In their study, the nanogenerator stimulated the movement of macrophages through interactions with cells and triggered the Saos‐2 ion channel in the plasma membrane of osteoblast‐like cells, inducing intracellular calcium transients. Additionally, this in situ ES of cells can be applied to other excitable cells (such as neurons or muscle cells).^[^ ^53^ ^]^ To address emergency hemostasis in skin wounds resulting from trauma, Meng et al. designed a bioadhesive triboelectric nanogenerator (BA‐TENG) (Figure 2i). This device uses polycaprolactone‐based polyurethane (PCL‐r‐PU) as a flexible top layer, with a bottom layer made of polyacrylic acid‐hydroxysuccinimide ester (PAA‐NHS) and polyvinyl alcohol (PVA), providing strong adhesion to wet tissues and an effective self‐powering capability. Simultaneously, the BA‐TENG also possesses ultrasound responsiveness and a strong electric field can be generated under ultrasound stimulation to accelerate skin wound healing, which is attributed to the enhancement of cell activity by the local electric field.^[^ ^54^ ^]^ In addition, Iman et al. designed an ultrasound‐driven triboelectric nanogenerator (IBV‐TENG) with subcutaneous antibacterial activity against infectious skin wounds. This nanogenerator utilizes triboelectrically positive poly‐3‐hydroxybutyric acid‐co‐3‐hydroxyvaleric acid (PHBV) as the encapsulation and active layers and polyvinyl alcohol (PVA) as the anti‐friction layer. It can respond to different ultrasound intensities to generate the corresponding electric fields and eliminate infectious microorganisms in subcutaneous tissues through ES. In both in vitro and in vivo experiments, the IBV‐TENG demonstrated excellent antibacterial properties. Beyond tissue repair and regeneration, nanogenerators hold significant potential in health‐monitoring applications.^[^ ^55^ ^]^ To alleviate the risk of diseases caused by long‐term abnormal postural habits, Li et al. developed a badge‐scroll‐shaped tensile sensor based on a grating‐structure TENG for real‐time monitoring and recording of joint movements. The seamless integration of flexible materials and TENGs endows this vector tensile sensor with numerous advantages such as wearability, conformality, and real‐time wireless monitoring, as well as excellent performance in spinal posture monitoring, contributing to a reduction in the risk of spinal diseases. In addition, this device has broad application prospects in other joint activity regions such as shoulders and ankles, offering a practical and feasible strategy for health detection and disease prevention.^[^ ^56^ ^]^

### 2.3 Piezoelectric Biomaterials

In recent years, piezoelectric biomaterials have attracted considerable attention in bioengineering because of their unique electricity‐generation properties and highly flexible material structures. These biomaterials provide a wide material platform for the miniaturization and automation of bioelectronic components and show extensive application prospects in sophisticated functional devices, such as flexible electronics and nanosensors.^[^ ^32^, ^57^, ^58^ ^]^ Therefore, this section summarizes and discusses the intrinsic and extrinsic piezoelectric characteristics of piezoelectric biomaterials and their applications in the biomedical field, and envisions the development prospects of these materials.

Piezoelectric biomaterials can spontaneously generate polarization, indicating that in certain specific application environments, sufficient electrical energy can be locally generated without an external power supply to sustain or modify the biological behaviors of tissues and cells, or to power microelectronic components.^[^ ^59^ ^]^ However, the miniaturization of piezoelectric components is difficult. Because of the reduction in size, the electric field generated by the piezoelectric effect is also weakened. Achieving an effective depolarization field at the micro‐nano‐scale is extremely challenging. In recent years, the emergence of novel, ultrathin piezoelectric materials has not only achieved considerable nano‐scale piezoelectric effects but also shown that the piezoelectric effect improves as material size decreases.^[^ ^24^ ^]^ With the in‐depth study of piezoelectric materials, synthesis methods have been continuously refined to meet market requirements. Currently, the mainstream synthesis methods for piezoelectric materials include chemical vapor deposition (CVD), electrochemical exfoliation, and mechanical micrometer‐scale exfoliation. These techniques work by overcoming interlayer adhesion or chemically producing single‐atomic‐layer nanosheets.^[^ ^60^ ^]^

**Figure 3:**
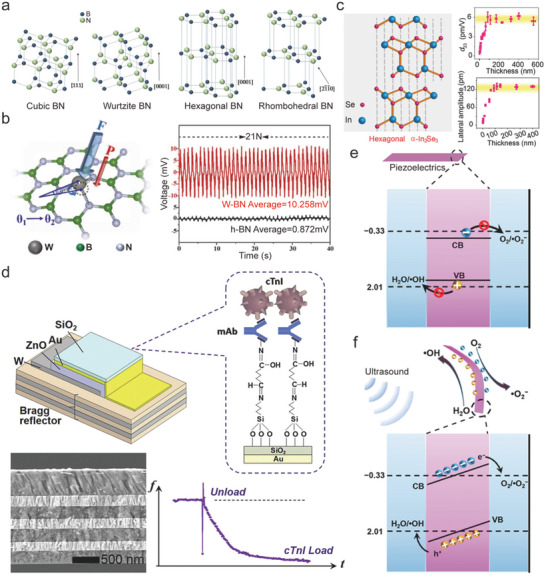
a) Crystal structures of cubic, wurtzite, hexagonal, and rhombohedral BN. Reproduced with permission.^[^ ^62^ ^]^ Copyright 2017, Wiley‐VCH. b) The anchoring of single‐atom tungsten (W) can cause significant lattice distortions in h‐BN lattice plane, thus introducing a strikingly enhanced piezoelectric response, approximately 12‐fold that of the parent h‐BN nanosheets. Reproduced with permission.^[^ ^68^ ^]^ Copyright 2022, Elsevier. c) Adopting stacking strategy, the corresponding *d*_33_ piezoelectric coefficient of α‐In_2_Se_3_ increases from 0.34 (monolayer) to 5.6 pm V^−1^ (bulk) without any odd–even effect. Reproduced with permission.^[^ ^70^ ^]^ Copyright 2018, American Chemical Society. d) A label‐free microfabricated thickness shear mode electroacoustic device based on a ZnO piezoelectric film for the assay of cardiac biomarkers. Reproduced with permission.^[^ ^80^ ^]^ Copyright 2020, Elsevier. e) and f): Piezoelectric materials as sonodynamic sensitizers to eliminate cancer cells with reactive oxygen species (ROS). Reproduced with permission.^[^ ^82^ ^]^ Copyright 2020, American Chemical Society.

A noncentrosymmetric lattice structure and non‐zero energy gap constitute two indispensable conditions that piezoelectric materials must satisfy. For example, hexagonal boron nitride (h‐BN) exhibits inherent piezoelectric attributes owing to its wide bandgap and noncentrosymmetric lattice configuration^[^ ^61^, ^62^, ^63^ ^]^ (**Figure** **3**a). Inspired by the natural structure of h‐BN, other hexagonal III–V semiconductors such as h‐GaN and h‐ZnO have also attracted the attention of scholars.^[^ ^64^, ^65^ ^]^ A common trait among them is that they possess two distinct, dynamically stable, 2D architectures: planar hexagons and wrinkled hexagons. Li et al. reported that mixed‐layer phase III–V compounds also exhibit piezoelectric properties.^[^ ^66^ ^]^ In addition, the asymmetric lattice structure of transition metal sulfide crystals (2h‐TMDC) also endows them with piezoelectric capabilities. However, the piezoelectric properties of most contemporary piezoelectric materials are poor. Hence, there is an urgent need to enhance the synthetic approaches to augment the piezoelectric performance of materials.

In modern industrial technology, there are numerous strategies for enhancing the piezoelectric properties of materials. One example is doping, where researchers introduce atoms such as Li, K, and F on one side of the graphene surface to disrupt the symmetry of its structure. This creates a noncentrosymmetric structure analogous to h‐BN, allowing graphene—previously nonpiezoelectric—to gain piezoelectric characteristics.^[^ ^67^ ^]^ Based on this principle, Zhang et al. loaded tungsten atoms on one side of h‐BN nanosheets to enhance the piezoelectric properties of the material.^[^ ^68^ ^]^ The experimental results suggested that even at an extremely low tungsten atom loading, the piezoelectric properties of the nanosheets enhanced by the doping strategy were significantly improved (approximately 34 times) compared to those of the original material (Figure 3b). Furthermore, doping transition metal sulfides to form Janus crystal structures is regarded as one of the most promising methods for inducing piezoelectric effects.^[^ ^69^ ^]^ Sulfur atoms selectively substitute certain atoms on the material surface to modify the overall crystal structure, resulting in the formation of z‐direction dipoles and the loss of z‐direction symmetry owing to variations in electron density. These crucial alterations further break the symmetry of the material lattice and increase structural heterogeneity, thereby enhancing the piezoelectric properties of the material.

In addition to doping, stacking is an effective method for improving piezoelectric performance. Stacked layers are classified as homogeneous or heterogeneous based on material characteristics. In homogeneous stacking, the piezoelectric effect depends on the monolayer's planar polarization direction. In AA stacking, adjacent layers have aligned polarization and dipole directions. Through this stacking strategy, multilayer structured materials, whether containing odd‐numbered or even‐numbered layers, can effectively exhibit piezoelectric properties and ultimately generate a robust piezoelectric effect. As the polarization directions between the layers are the same, the piezoelectric coefficient of the overall material is proportional to the number of stacked layers^[^ ^70^ ^]^ (Figure 3c). In contrast, the dipole directions between the adjacent layers of multilayer materials in AB stacking are opposite, and the piezoelectric effect of each monolayer is neutralized by the adjacent layer. Therefore, the overall piezoelectric output mainly depends on the piezoelectric effect of a few layers.^[^ ^33^ ^]^ In contrast, heterogeneous stacking is advantageous in practical applications. Alternately stacked piezoelectric polymers with nano‐materials, such as metal oxides, metal nanoions, and nanotubes, to form heterogeneous structures can effectively enhance the piezoelectric properties of composite materials. PVDF, one of the most common piezoelectric polymers, is extensively used in biological research and medical consumables. During the production of PVDF films, doping different proportions of ZnO nanoparticles during electrospinning can improve the piezoelectric properties of composite films to varying degrees.^[^ ^71^ ^]^ This is attributed to the embedding of ZnO, which modifies the crystal structure of PVDF, increases the proportion of the β‐phase lattice, and further generates a synergistic effect with PVDF nanofibers, resulting in a remarkable enhancement in the piezoelectric properties of the overall composite material.^[^ ^72^ ^]^

For inorganic bio‐piezoelectric materials, modifying the lattice size of the material itself is an effective approach for enhancing piezoelectric performance. Conventionally, it is widely recognized that a high surface‐area ratio is a critical factor that triggers surface effects. Thus, when the scale of a material reaches the nanometer or sub‐nanometer level, the performance of the piezoelectric material is further enhanced, and even nonpiezoelectric materials may exhibit piezoelectric properties. For instance, when the lattice size of BaTiO_3_ ceramics approaches 1 µm, a significant improvement in piezoelectric performance occurs. Additionally, researchers have augmented the piezoelectric constant of the material by reducing the diameter of the BaTiO_3_ nanofibers.^[^ ^73^ ^]^

Biocompatibility is another parameter that cannot be overlooked when bio‐piezoelectric materials are utilized in the biomedical field. Biosecurity is a core factor that determines whether bio‐piezoelectric materials can pass tests and be practically applied in biomedicine. For example, lead zirconate titanate (PZT) is a piezoelectric material with remarkable piezoelectric performance. However, as the material contains lead (Pb), which is a heavy metal with biological toxicity, it cannot be directly applied to cells or tissues. Researchers have mitigated the cytotoxicity of this material by conducting surface modifications or applying a biological coating to PZT, exerting its excellent piezoelectric properties while enhancing the biosafety of the material.^[^ ^74^ ^]^ The hydrophilicity of a material, which is an important indicator of bioadhesion, has received considerable attention. PVDF films are unfavorable for cell adhesion and proliferation because of their high surface hydrophobicity. Kitsara et al. performed surface modification of a fiber membrane through plasma treatment to form a long‐term stable superhydrophilic layer on the surface of the fiber membrane, resolving the problem of cell adhesion. In addition to the hydrophilicity of the piezoelectric material surface, cell adhesion depends on its roughness. The smooth surface of PCL piezoelectric scaffolds is detrimental to cell adhesion, but incorporating ZnO nanoparticles increases roughness, improving cell adhesion and enhancing piezoelectric performance.^[^ ^75^ ^]^

Through continuous research, many new piezoelectric materials have been developed and widely applied in medicine.^[^ ^76^, ^77^ ^]^ Since their introduction in 1962, biosensors have steadily evolved toward miniaturization, with bio‐piezoelectric materials providing crucial support. Traditional biosensors struggle with portability and wearability due to their large size and reliance on external power. In contrast, piezoelectric biomaterials, with their excellent piezoelectric properties, biocompatibility, and micro‐nano‐scale, enable biosensors to be implantable, highly sensitive, and self‐powered. Additionally, polymer piezoelectric materials endow biosensors with outstanding flexibility, allowing them to adhere impeccably to tissues and organs, thereby enhancing the accuracy of monitoring results. In recent years, a series of wearable, health‐monitoring platforms based on flexible bio‐piezoelectric materials have attracted considerable attention. For instance, based on the exceptional piezoelectric properties and flexibility of PVDF nanofiber membranes, Liu et al. designed a wearable respiratory detector that continuously outputs electrical signals through the deformation of a PVDF piezoelectric film during breathing. Simultaneously, the high piezoelectric coefficient of the PVDF fiber membrane ensures the high sensitivity of the respiratory detector.^[^ ^78^ ^]^ Mao et al. integrated T‐ZnO nanowires into textiles and developed a dynamic monitoring sensor for various body parts. Through stretching of the textile by skin tissue during daily activities, T‐ZnO nanowires can convert mechanical energy into electrical signals to continuously monitor and sense physiological activities.^[^ ^79^ ^]^ Beyond sensing dynamic signals on the body surface, bio‐piezoelectric materials offer an optimal solution for disease diagnosis. Liu et al. developed a microdetector using ZnO piezoelectric fiber membranes to detect myocardial necrosis markers, such as cardiac troponin, for the early prevention of acute cardiomyopathy. The device's high sensitivity is attributed to the excellent piezoelectric properties of the ZnO fibers^[^ ^80^ ^]^ (Figure 3d). Furthermore, bio‐piezoelectric probes based on piezoelectric materials have considerable application prospects in tumor diagnosis. For example, the bioprobe developed by Su et al. based on PZT ceramics can realize the quantitative detection of tumor markers in serum by responding to the interaction between antigens and antibodies.^[^ ^74^ ^]^ In addition to monitoring tumor molecular signals, bio‐piezoelectric probes can diagnose tissue lesions through changes in mechanical properties such as tissue texture and density.^[^ ^81^ ^]^

Bio‐piezoelectric materials are also playing an increasingly significant role in disease treatment. Bio‐piezoelectric materials generate considerable quantities of reactive oxygen species (ROS) by electrically stimulating tumor cells. Simultaneously, electrical signals regulate the proliferation and metabolism of tumor cells, ultimately achieving the goal of killing tumor cells. Li et al. developed an acoustic dynamic tumor treatment sensitizer based on piezoelectric BP nanosheets. Both in vitro and in vivo experiments demonstrated that this material could induce tumor cells to produce ROS and effectively inhibit their proliferation of tumor cells^[^ ^82^ ^]^ (Figure 2e,f). Feng et al. exerted mechanical stress on nano/micrometer tetragonal‐BaTiO_3_, which caused the nanoparticles to generate unbalanced charges and further stimulated cells or bacterium to produce a large amount of ROS, leading to death.^[^ ^83^ ^]^ Furthermore, piezoelectric biomaterials, characterized by their ability to convert mechanical energy into electrical energy, have shown great promise in non‐invasive electrical stimulation therapies, particularly in the fields of nerve repair, bone tissue regeneration, wound healing, and cardiovascular disease treatment.^[^ ^84^ ^]^ The core mechanism lies in the generation of localized electric fields by these materials, which can significantly regulate cell proliferation, differentiation, migration, and functionality, thereby supporting tissue repair and regeneration.^[^ ^85^ ^]^ In nerve repair, piezoelectric materials generate electrical potentials in response to mechanical stimuli, modulating the membrane potential of nerve cells and activating voltage‐gated sodium (Nav) and potassium (Kv) channels to facilitate the generation and transmission of action potentials. This effect not only guides synaptic formation but also induces the secretion of neurotrophic factors, such as brain‐derived neurotrophic factor (BDNF), further supporting nerve network reconstruction.^[^ ^86^, ^87^ ^]^ For instance, PVDF fiber conduits generate sustained electrical stimulation from micro‐movements, accelerating sciatic nerve repair in animal models and improving nerve conduction velocity. Similarly, nanocomposites containing barium titanate have demonstrated enhanced synaptogenesis and electrical signal transmission in vitro.^[^ ^88^, ^89^ ^]^ In bone tissue regeneration, piezoelectric materials mimic the natural piezoelectric effect of bones, playing a pivotal role in bone repair. Mechanical loading‐induced electric fields activate calcium ion channels, such as TRPV4, and the bone morphogenetic protein (BMP) signaling pathway, promoting the proliferation and differentiation of osteoblasts.^[^ ^76^, ^90^ ^]^ Furthermore, these fields enhance extracellular matrix (ECM) mineralization and regulate the expression of adhesion proteins like integrin α5β1, accelerating bone tissue remodeling.^[^ ^91^ ^]^ Research on PZT nanoparticles has shown significant improvements in mineralization efficiency in bone marrow‐derived mesenchymal stem cells (BMSCs), while PLLA piezoelectric scaffolds have demonstrated increased bone density and angiogenesis in fracture models. These dual functions make piezoelectric materials uniquely suited for bone repair applications.^[^ ^92^ ^]^ In wound healing, piezoelectric materials create micro‐electric fields that act on keratinocytes and fibroblasts, activating the PI3K/AKT signaling pathway to enhance cell proliferation and migration.^[^ ^93^ ^]^ Additionally, the electric currents induced by piezoelectric effects stimulate the secretion of vascular endothelial growth factor (VEGF), promoting angiogenesis to improve the microenvironment with better oxygen and nutrient supply. Piezoelectric stimulation also modulates macrophage polarization around the wound, suppressing excessive inflammation and accelerating tissue reconstruction.^[^ ^90^ ^]^ For example, flexible PVDF sensors that generate weak electric fields from dynamic movements have significantly reduced healing time in skin wound models.^[^ ^94^ ^]^ Piezoelectric antibacterial dressings incorporating silver ions further reduce infection risks while expediting wound closure.^[^ ^95^ ^]^ In cardiovascular disease treatment, piezoelectric materials provide innovative solutions by generating dynamic electric fields to regulate cardiomyocyte and endothelial cell behavior. These fields stabilize calcium ion flux and activate relevant signaling pathways, improving the synchronized contraction and electrical conductivity of cardiomyocytes, thereby enhancing heart function. Moreover, dynamic electric stimulation promotes VEGF secretion, facilitating vascular repair and neovascularization, particularly in ischemic tissues.^[^ ^90^ ^]^ Flexible piezoelectric films applied to cardiomyocyte cultures have significantly improved rhythmic contraction and functional integration.^[^ ^96^ ^]^ Meanwhile, vascular stents incorporating barium titanate cores have shown excellent results in promoting endothelial cell proliferation and vascular regeneration in animal models.^[^ ^97^ ^]^ Furthermore, piezoelectric materials are being explored for self‐powered cardiac pacemakers, harvesting biomechanical energy from body movements to provide sustained electrical stimulation, thereby reducing dependence on battery replacements.^[^ ^98^ ^]^ Piezoelectric biomaterials, with their unique ability to deliver electrical stimulation, interact synergistically with various cell types to support the repair and regeneration of nerve, bone, skin, and cardiovascular tissues. As material designs are optimized and biological mechanisms are further elucidated, these materials hold immense potential for clinical translation and broader applications in regenerative medicine.

### 2.4 Triboelectric Scaffolds

Currently, there are two main types of triboelectric materials: electrospun triboelectric materials and cellulose triboelectric materials. The electrospinning process for fabricating triboelectric offers exceptional control over the micro‐nano‐scale surface structure, allowing for precise adjustments to surface roughness to meet specific requirements. Compared to manufacturing techniques such as the template method, self‐assembly, and 3D printing, electrospinning has advantages such as low cost, ease of operation, and high controllability, fulfilling the demands of large‐scale batch production.^[^ ^99^ ^]^

The principal forms of electrospun triboelectric materials include nanofibers with different orientations (random and directional), microspheres with rough surfaces (e.g., spherical and honeycomb‐like), and hierarchical structures (e.g., porous and core–shell nanofibers). Among these, random nanofibers are the most widely used in triboelectric materials.^[^ ^100^, ^101^ ^]^ Owing to their high surface‐area ratio and surface roughness, random nanofibers are frequently utilized as friction layers to enhance the triboelectric properties of materials. In contrast, directional nanofibers are spun in a predefined direction during the electrospinning process, thereby generating an anisotropic triboelectric performance. Wang et al. obtained a triboelectric film through directional electrospinning of PVDF nanofibers that could output electrical signals of different intensities when exposed to friction stress in different directions.^[^ ^102^ ^]^ However, numerous challenges remain in the preparation of nanofiber films via electrospinning. Parameters such as the molecular weight of the polymer, concentration of the precursor solution, and magnitude of the spinning voltage can significantly influence the triboelectric performance of the nanofiber membrane.^[^ ^103^, ^104^, ^105^, ^106^, ^107^ ^]^ Low‐molecular‐weight polymers result in the formation of beaded fibers or even an inability to form fibers owing to insufficient entanglement. An excessively low concentration of the precursor solution causes spraying at the electro‐nozzle instead of electrospinning. Conversely, an overly high concentration is prone to blockage at the exit of the electro‐nozzle and fails to form continuous and uniform spun fibers. The electric field intensity applied during electrospinning directly determines the diameter of the fibers. Excessive voltage is not conducive to the formation of continuous and uniform nanofibers and is highly likely to cause beaded fibers or fiber fractures owing to excessive ejection speed. Additionally, for porous triboelectric materials, such as random nanofibers, porosity plays a key role in triboelectric performance. The electrospinning process allows for fine control over pore size between nanofibers.

Electrospraying technology is the most widely utilized procedure for the manufacture of electrospun microspheres and constitutes a variation of electrospinning technology. Factors such as an excessively low molecular weight of the polymer resulting in insufficient entanglement among molecular chains, an overly low concentration of the precursor solution causing inadequate solution viscosity, or an overly large electric field intensity applied during the electrospinning process all lead to the discontinuity of the jet during the spinning process, thereby forming particles of various shapes.^[^ ^108^ ^]^ By further adjusting experimental parameters, electrospun microspheres with uniform and controllable particle sizes can be fabricated. Compared to electrospun nanofibers, electrospun microspheres have a larger surface‐area ratio and surface roughness. Consequently, electrospun microspheres exhibit superior triboelectric properties. However, owing to the mechanical properties that require improvement, there are currently relatively few cases of individual applications of electrospun microspheres. They are mainly employed as filling materials in the preparation of composite triboelectric materials to enhance their triboelectric properties or hydrophobicity.^[^ ^109^, ^110^ ^]^

Layered‐structured nanofibers are 2D or 3D structures based on electrospun nanofibers, which are mainly categorized into porous nanofibers and core–shell nanofibers.^[^ ^111^, ^112^ ^]^ The fundamental principle of preparing porous nanofibers through electrospinning lies in separating solvents, blends, and polymers during the fabrication process by exploiting the differences in solubility and volatility and forming pores on the surface and within the polymer fibers. Commonly adopted methods include gas phase separation, nonsolvent phase separation, thermally induced phase separation, and template sacrifice.^[^ ^113^, ^114^, ^115^ ^]^ By regulating the pore size and quantity, triboelectric materials with outstanding hydrophobicity and electrical properties can be fabricated to satisfy diverse application requirements. Core–shell‐structured nanofibers are obtained by preparing two immiscible or semi‐miscible polymers into nanofibers with a core–shell structure via a coaxial electrospinning process.^[^ ^116^ ^]^ The concentration, viscosity, compatibility, flow rate ratio, and spinning voltage of the two‐phase solution are all crucial factors influencing core–shell nanofibers. Notably, the flow rate of the core phase solution must be lower than that of the shell phase solution to ensure the integrity and continuity of the shell structure during the spinning process.

Another major category of triboelectric materials is cellulose. Cellulose is widely present in plants and an abundant source of raw materials. As a natural and environmentally friendly material, cellulose exhibits excellent biocompatibility, degradability, and mechanical strength.^[^ ^117^, ^118^, ^119^ ^]^ Its molecules contain a large number of modifiable hydroxyl reactive groups and can be modified to produce triboelectric materials such as hydrogels, aerogels, films, and fabrics.^[^ ^120^, ^121^, ^122^, ^123^ ^]^ Researchers have effectively enhanced the triboelectric properties of materials by increasing the interface area and improving the dielectric constant of composite materials through different interface design strategies, thereby enhancing charge density or regulating charge characteristics.

The extraction of nanocellulose from diverse raw plant materials is a top‐down process. Mechanical grinding and homogenization are the most widely used physical methods. The combination of chemical or biological approaches, such as acid and enzymatic hydrolysis, can further disintegrate the intertwined regions within nanocellulose, thereby enhancing the specific surface area or relative crystallinity of the material.^[^ ^124^, ^125^, ^126^ ^]^ Additionally, techniques such as chemical modification or ultrasound can regulate the surface charge of nanocellulose and subsequently control ion transport.^[^ ^127^ ^]^ Theoretically, this indicates that nanocellulose‐based flexible electrical materials facilitate energy collection and storage, thereby presenting extensive application prospects. In contrast to the aforementioned processes, the synthesis of cellulose materials from nanocellulose is a bottom‐up process and constitutes a preparation approach for cellulose‐based triboelectric materials. Additionally, carbonizing nanocellulose into graphene improves its electrical properties, while the porous network structure of cellulose aids electron and ion transmission, providing a solid foundation for energy transmission and storage.^[^ ^128^ ^]^ For cellulose composite triboelectric materials, the degree of dispersion among nanofibers has a decisive influence on the performance and service life of the materials. Moreover, in water, the degree of dispersion of nanofibers can be accomplished by regulating the surface charge of the fibers. Therefore, the appropriate introduction of charges during the preparation of cellulose composite materials can increase the electrostatic repulsion between nanocellulose molecules, thereby fabricating a uniformly dispersed fiber membrane.^[^ ^129^ ^]^

With advancements in the manufacturing process and performance optimization of triboelectric materials, more electronic products are being applied in healthcare. Wearable garments made from triboelectric materials can harness kinetic energy from joint and muscle movements during daily activities, converting and storing it as electrical energy.^[^ ^130^ ^]^ For example, a microsensor based on a composite triboelectric material of microcrystalline cellulose and polyvinyl alcohol could effectively harvest energy during human movement when adhered to the sole of the foot.^[^ ^131^ ^]^ Electrospun nanofiber‐based electronic skin adheres comfortably to human skin, even during extended wear, enabling real‐time monitoring of human health indicators such as breathing and heartbeat by sensing skin expansion and contraction.^[^ ^132^ ^]^

### 2.5 Zwitterionic Hydrogels

Zwitterionic hydrogels are gel materials formed by cross‐linking zwitterionic polymers. They exhibit unique ionic conductivity, favorable biocompatibility, and high hydrophilicity, effectively preventing nonspecific adsorption of proteins and adhesion of microorganisms, such as bacteria.^[^ ^133^, ^134^, ^135^ ^]^ Given these properties, zwitterionic hydrogels, as flexible materials with electrical characteristics, have broad application prospects in biomedicine.

Zwitterionic hydrogels can be classified into four types based on chemical components and preparation methods.^[^ ^136^, ^137^ ^]^ (I) Single‐network pure zwitterionic hydrogels are single‐network polymer hydrogels formed by cross‐linking cationic and anionic monomers. While they have excellent biocompatibility, their low cross‐linking and lack of mechanical dissipation result in poor mechanical performance, limiting their independent use in practical applications. (II) Zwitterionic copolymer hydrogels include supramolecular copolymer zwitterionic hydrogels and in situ crosslinked zwitterionic hydrogels. Supramolecular hydrogels are created by copolymerizing zwitterionic monomers with oppositely charged polymers in a 1:1 ratio, resulting in a uniform charge distribution and electrically neutral material. In situ crosslinked hydrogels, on the other hand, are formed by grafting zwitterionic side chains onto alkenyl polymer main chains. These hydrogels are rich in functional groups, allowing for further modifications to introduce additional properties tailored to diverse application requirements. (III) Zwitterionic hydrogels modified with natural polymers are obtained by introducing zwitterionic monomers into natural polysaccharides via chemical reactions. They exhibit good biocompatibility and degradability and are applicable in biomedical fields such as in vivo implantation and drug delivery. (IV) Multinetwork zwitterionic hydrogels are fabricated by adopting double‐ or triple‐network strategies. Their multinetwork structures endow them with outstanding mechanical properties.

Driven by an external electric field, the cations and anions within zwitterionic hydrogels tend to separate and undergo directional movement, thereby giving rise to charge transfer and the formation of an electric current.^[^ ^138^, ^139^, ^140^ ^]^ Consequently, zwitterionic hydrogels exhibit excellent electrical conductivity. Simultaneously, cationic and anionic groups interact with water molecules to form robust hydrogen bonds and establish a dense hydration layer on the surface of the material. This electrostatically induced hydration confers zwitterionic hydrogels with favorable antifouling properties and enables them to effectively resist the nonspecific adsorption of proteins and adhesion of microorganisms, such as bacteria.^[^ ^141^, ^142^ ^]^ This feature provides a favorable material foundation for the antifouling performance of wearable devices. In addition, zwitterionic hydrogels with various response properties can be fabricated by introducing diverse functional groups into the hydrogel network.

Owing to their remarkable electrical properties and interfacial characteristics, zwitterionic hydrogels have broad application prospects in the biomedical field. Xu et al. constructed a tumor marker sensor based on polydopamine (PDA) and poly (sulfobetaine methacrylate) to detect tumor marker content in serum samples.^[^ ^143^ ^]^ Xie et al. developed a sensor with a zwitterionic polymer coating to eliminate interference signals initially generated during the implantation of a continuous glucose monitor. The results indicated that this sensor effectively reduced signal noise and enhanced the accuracy of blood glucose detection.^[^ ^144^ ^]^ Moreover, researchers prepared reusable zwitterionic polymer hydrogels with outstanding mechanical properties based on acrylic acid, octadecyl methacrylate, and sulfobetaine methacrylate (SBMA). Benefiting from the remarkable strain sensitivity of this material, it can be employed not only to monitor limb movements but also to monitor subtle movements related to pulse and breathing.^[^ ^145^ ^]^ Although current research on zwitterionic hydrogels is burgeoning, there is still a considerable gap in their industrial mass production. Numerous challenges, such as limited polymer availability, poor chemical stability, and insufficient mechanical strength, still need to be addressed.

### 2.6 Biosafety Evaluation of Electrical Biomaterials

The majority of electrical biomaterials exhibit commendable in vitro biocompatibility. For example, piezoelectric materials derived from polylactic acid (PLA) or polyhydroxy butyrate (PHB) demonstrate relatively low cytotoxicity.^[^ ^91^ ^]^ Triboelectric scaffolds have been shown to enhance cell adhesion and proliferation through electrical stimulation, facilitating the differentiation of osteoblasts and fibroblasts.^[^ ^146^, ^147^ ^]^ Zwitterionic hydrogels possess favorable ion‐regulating capabilities that help maintain ionic balance both intracellularly and extracellularly.^[^ ^148^, ^149^ ^]^ Nanogenerators and zwitterionic hydrogels generally elicit mild immune or inflammatory responses in animal studies, primarily linked to the surface modifications and degradation products of these materials.^[^ ^150^ ^]^ When implanted into bone defect models, piezoelectric materials effectively promote bone tissue regeneration without inducing significant long‐term inflammation.^[^ ^19^, ^151^ ^]^ Detailed information regarding the in vivo concentration/dose, biodegradation profiles, and long‐term biosafety of various electrical biomaterials is presented in **Table** **1**.

**Table 1:** Biosafety evaluation of electrical biomaterials.

Electrical biomaterials	Usage concentration/dosage	Degradation products	Degradation time	Biocompatibility	Long‐term safety evaluation
Nanogenerators	Micrograms to milligrams	Zn^2^⁺ and other ions	Days to weeks	Good (Zn^2^⁺ in moderation promotes cell function)	Excess Zn^2^⁺ may lead to toxicity; chronic safety needs evaluation
Piezoelectric Materials	Hundreds of milligrams to grams	Small molecules like lactic acid	Weeks to months	Excellent (promotes bone tissue repair)	Long‐term stability, no significant rejection observed
Triboelectric Scaffolds	10–50 mg/scaffold	Small organic acids	1–6 months	Good (significant tissue repair promotion)	Scaffold properties need to match tissue to avoid overstimulation
Zwitterionic Hydrogels	5–10% (w/v)	Controlled hydrolysis products	Days to months	Excellent (good ion regulation capability)	Long‐term degradation product metabolism and immune response need evaluation

## 3 Applications of Electrical Biomaterials for Bone and Cartilage Regeneration

### 3.1 Electric Stimulation for Bone Regeneration

**Figure 4:**
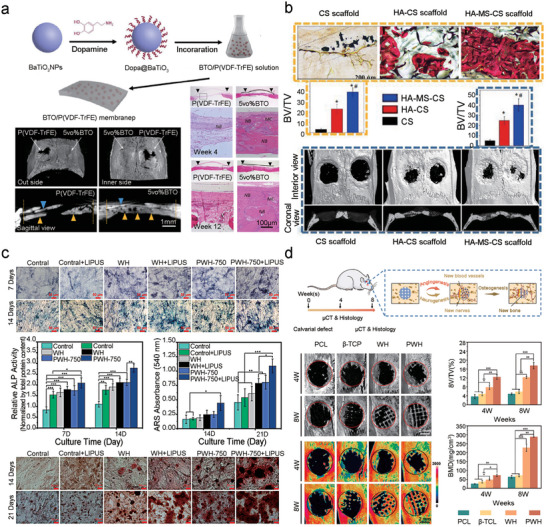
The applications of piezoelectric materials and nanogenerators for bone regeneration. a) BTO/P(VDF‐TrFE) film exhibited a favorable bone healing in rat calvarial models. Reproduced with permission.^[^ ^163^ ^]^ Copyright 2016, American Chemical Society. b) Histological outcomes and Micro‐CT image of bone healing calvarial models with CS, HA‐CS and HA‐MS‐CS implant show more newly developed bone tissues compared with HA‐CS and CS scaffold in vivo.^[^ ^159^ ^]^ Copyright 2017, American Chemical Society. c) WH NPs and PWH‐750 NPs exhibit different osteogenic differentiation potential with or without LIPUS treatment.^[^ ^152^ ^]^ Copyright 2021, Elsevier. d) Biomimetic piezoelectric scaffolds promote nerve and vascular regeneration via continuously releasing Mg^2+^ to enhance bone regeneration.^[^ ^157^ ^]^ Copyright 2023, KeAi Communications Co.

Bone possesses essential bioelectric properties that are vital for the remodeling process, and its degradation under various pathological conditions results in significant alterations in these properties. Researchers are increasingly focusing on the utilization of biomimetic electroactive materials to enhance bone repair by mimicking the natural electrophysiological environment of healthy bone tissue. Theoretically, bone mass and strength rely on a balance between osteoclast‐mediated resorption and osteoblast‐driven formation. Regulating the electric field within the bone microenvironment through microdevices can accelerate bone formation and offer effective treatment for injuries. EnEFs in bones also play a role in the regulation of cellular metabolism, including growth, proliferation, differentiation, and motility (**Figure** **4**c).^[^ ^152^, ^153^ ^]^ For instance, during walking, human tibia generates a piezoelectric potential of ≈300 µV.^[^ ^91^ ^]^ Natural bioelectrical signals from living bones, such as piezoelectricity, pyroelectricity, and ferroelectricity, are recognized as key factors influencing metabolic processes such as growth regulation, structural remodeling, and fracture healing.^[^ ^5^, ^12^ ^]^

Bone tissue consists of inorganic minerals and organic collagen, with the collagen/HA complex imparting piezoelectric properties to bone tissue. Utilizing piezoelectric materials to recreate the physiological electrical microenvironment at defect sites is an effective approach for enhancing osteogenesis, particularly during the early stages. Biomimetic scaffolds that replicate both the composition and piezoelectric characteristics of bones are promising candidates for regenerative medicine. The noncentrosymmetric nature of collagen molecules has been identified as a key factor contributing to bioelectricity in healthy bones. In addition, piezoelectric collagen within the bone generates a streaming potential when subjected to stress, which leads to decreased hydraulic permeability and increased stiffness. Kwon proposed a perspective on ACP infiltration that emphasizes the physiological significance of collagen piezoelectricity in intrafibrillar mineralization.^[^ ^154^, ^155^, ^156^ ^]^ Yu et al. developed a biomimetic piezoelectric composite scaffold with a sustained release of magnesium ions. By incorporating magnesium calcium phosphate, scaffolds gain electrical properties similar to bone, thereby enhancing osteogenesis (Figure 4d).^[^ ^157^ ^]^ Apart from piezoelectricity, the biological response of hard tissues can be improved by direct electrical stimulation.^[^ ^158^ ^]^ Sun et al. synthesized hydroxyapatite nanowire@magnesium silicate nanosheet (HANW@MS) core–shell porous hierarchical nanocomposites (nanobrushes) and implanted chitosan (CS), HA‐CS, and HA‐MS‐CS in bone defect areas. They performed histological staining and found that compared with HA‐CS and CS scaffolds, HA‐MS‐CS scaffold exhibited enhanced bone repair. Micro‐CT images of the defect area showed that the bone growth of the group implanted with a piezoelectric MS based stent (HA‐CS‐MS) was significantly better than that of the control stent group without MS (i.e., CS, HA‐CS). Coronary and internal 3D views showed that bone growth was qualitatively and quantitatively enhanced after implantation of the piezoelectric MS (HA‐CS‐MS) stent (Figure 4b).^[^ ^159^ ^]^ Thrivikraman et al. explored how physical factors, such as surface elasticity, topography, and chemistry, affect mesenchymal stem cell (MSC) behavior, showing that a combination of substrate conductivity, chemical composition, and electric pulses can direct MSC differentiation toward osteogenesis.^[^ ^160^ ^]^ Basu et al. developed a theory suggesting that bioelectric stress can alter cell morphology and electroporation, thus influencing the overall cell response to electric fields in the context of biomaterials.^[^ ^161^ ^]^ However, selecting appropriate piezoelectric materials to meet the different requirements of the regeneration process remains challenging.

The stress‐induced potential in piezo‐bioceramics and piezobiopolymers augments bone metabolism (Figure 4a).^[^ ^162^, ^163^ ^]^ Piezoelectric bioceramics have proven to be an effective in vivo energy source for biosensors and pacemakers. Wang et al. analyzed the power generation characteristics of PZT hard ceramics and their equivalent circuits through extensive experiments.^[^ ^164^ ^]^ McKinstry et al. synthesized a two‐step pyrolysis solution to produce thin films with dense columnar microstructures, which is significant for orientation control and enhancement of piezoelectric performance. This new solution exhibited good dielectric, ferroelectric, and breakdown field characteristics.^[^ ^165^ ^]^ Other studies have reported that piezoelectric bioceramics/biopolymers generate surface charges under external stress similar to those in bones. Notably, polarized piezoelectric surfaces can improve the osteogenic performance of bones. To address the issue of aseptic loosening of bone implants, Zhou et al. designed a surface coating of implants based on piezoelectric poly (l‐lactic acid) (PLLA).^[^ ^14^ ^]^ The scaffold was endowed with bone‐like mechanical strength and a conducive chemical environment through a controllable mineralization strategy facilitated by piezoelectric catalysis on the surface of PLLA fibers. Additionally, the PLLA fibers preserved their piezoelectric signal, which could effectively stimulate stem cells to differentiate into osteoblasts in response to ultrasound‐activated electrical signals, thereby promoting bone regeneration.

Piezoelectric implants enhance fracture repair by generating ES at the injury site through physiological stress. This hyperpolarization activates voltage‐gated calcium channels in the cell membrane, with intracellular Ca^2+^ playing a critical role in cell proliferation. Consequently, biocompatible piezoelectric scaffolds can promote early‐stage tissue repair more effectively than nonpiezoelectric ones.^[^ ^166^ ^]^ Liu et al. compared the bone integration of positively polarized BiFeO_3_ membrane strontium titanate (STO), negatively polarized BFO membrane STO implants, and nonpolarized STO implants fixed in rat femurs. The electric field at the interface between +BFO‐STO (+75 mV) and the negatively charged bone defect surface (−52 to −87 mV) enhanced bone integration compared to –BFO‐STO and nonpolarized STO surfaces.^[^ ^167^ ^]^ In a separate study, composite ceramics made from barium titanate (BT; BaTiO_3_,) and hydroxyapatite (HA) were used as dielectric materials and 3D‐printed using digital light processing technology to create BT/HA bone scaffolds. These 3D‐printed scaffolds exhibited excellent biological and dielectric properties, force sensitivity, and biocompatibility, and were found to shorten bone regeneration time. The combination of these properties suggests that ceramic piezoelectric composites could be a new generation of bone regeneration implants. This study also explored the development of artificial bone materials with piezoelectric properties using varying ratios of BT and HA. Composite samples were prepared via compression molding and characterized for their mechanical, electrical, and biological properties, meeting the demands for mechanical strength, biological compatibility, and piezoelectric performance. The results indicated that the mixture of 95wt% BT and 5wt% HA exhibited excellent mechanical properties and biological activity. In the future, this piezoelectric bone material could be used as a 3D printing consumable, combined with 3D printing technology, to achieve personalized customization of human bones, providing an important reference for the current clinical challenges of artificial bone defects.

Efforts to treat bone nonunion with ES date back to 1812, but significant interest in the relationship between bone and electricity only emerged after the discovery of bone's piezoelectric properties in 1953.^[^ ^168^ ^]^ ES proved effective in accelerating new bone formation. However, the need for an external power source limited its portability and real‐time application. The emergence of friction nanogenerators (i.e., TENGs) in 2012 revitalized the field by enabling small, wearable energy‐harvesting devices.^[^ ^169^ ^]^ TENGs can convert micro‐ and nano‐scale energy sources, such as heartbeats, pulses, and walking, into electrical energy. By harnessing the mechanical energy of human activities, TENGs could provide a wearable, self‐powered method for electrically stimulating bone formation and regeneration.

**Figure 5:**
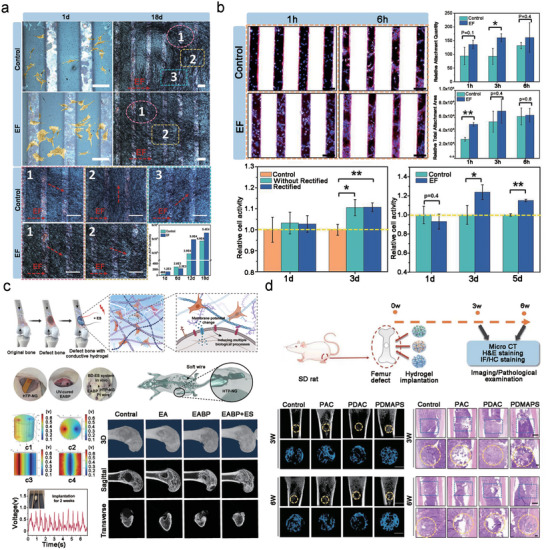
The applications of zwitterionic hydrogel and triboelectric scaffold for bone regeneration. a) Self‐powered electrical stimulator promotes adhesion and proliferation of osteoblasts.^[^ ^172^ ^]^ Copyright 2019, Elsevier. b) Self‐powered electrical stimulator promotes osteogenesis of MC3T3‐E1 cells.^[^ ^172^ ^]^ Copyright 2019, Elsevier. c) Hybrid tribo/piezoelectric nanogenerator for repairing bone defects through self‐powered electrical stimulation.^[^ ^12^ ^]^ Copyright 2024, AAAS. d) An implantable poly zwitterionic hydrogel scaffold (PDMAPS) was synthesized using methacrylated sulfobetaine (DMAPS) and acrylated gelatin (GelMA) as the matrix. Using polyanionic fiber (PAC) and polydimethyldiallylammonium chloride (PDAC) as control, the repairing effect of PDMAPS hydrogel scaffold on rat femoral defect was observed.^[^ ^179^ ^]^ Copyright 2023, Wiley‐VCH.

Zhou et al. focused on the application of TENGs in biomedicine. In 2014, they first used the electrical energy generated by rat respiration to drive a pacemaker. In 2016, they successfully converted the mechanical energy generated by the heartbeat into electrical energy, achieving self‐driven, real‐time monitoring of the heart rate over a long range.^[^ ^170^, ^171^ ^]^ Furthermore, in response to the repair characteristics of bone and joint injuries, Zhou et al. designed a self‐driving ES device based on the basic principle of TENGs and conducted in vitro ES experiments using pre‐osteoblast MC3T3‐E1 (**Figure** **5**a,b).^[^ ^172^ ^]^ Through in vitro experiments, they demonstrated that the constructed self‐driving ES device promoted the adhesion, proliferation, and differentiation of osteoblasts. They found that after 3 h of ES, the cell adhesion rate increased by 72.76% compared with that in the control group, and after 3 d of ES, the cell proliferation rate increased by 23.82%. In addition, the flexible TENG was implanted on the femoral surface of mice, where it successfully generated electrical signals during leg movements. This confirmed its potential for in situ stimulation, offering a new approach for clinical fracture treatment and bone remodeling.^[^ ^173^ ^]^ Wang et al. reported an intelligent “tissue battery” utilizing friction nanogenerators for real‐time monitoring of cartilage repair. This device used self‐generated microcurrents to stimulate rapid cartilage repair and provided real‐time visualization of the repair process, integrating tissue regeneration with real‐time monitoring (Figure 5c).^[^ ^12^ ^]^

Early TENG materials, including 2D materials and nanoparticles, faced challenges such as mechanical damage, high costs, limited sustainability, and complex manufacturing processes.^[^ ^174^ ^]^ In contrast, ion layers can be customized using various materials, making them promising solutions for TENG interlayer materials. Different materials have been used as intermediate layers to improve the performance of TENGs. However, challenges persist in terms of cost and stability. Previous studies have primarily focused on the electrical performance of TENGs and have often neglected their mechanical properties. However, sacrificing mechanical performance to improve TENG output performance may limit their potential in various flexible biological devices. Therefore, adding an ionic liquid‐based double layer (EDL) to TENGs is a favorable approach for improving output performance by reducing frictional charge decay without affecting mechanical durability.^[^ ^175^ ^]^ Kim et al. proposed the use of ionic electrolyte polymers as intermediate layers. This method effectively reduces charge decay and maximizes TENG efficiency by utilizing the high charge capacitance of an ion double layer (iEDL). This device exhibited significant stability over 10 000 operating cycles and effectively maintained its performance when subjected to external physical forces, such as cuts, penetrations, and severe creases caused by blades.^[^ ^175^ ^]^ Other researchers developed stretchable TENGs based on micropatterned fabric‐coated molybdenum disulfide (MoS) and Ecoflex nanocomposites as electron acceptor layers for wearable biomechanical energy‐harvesting and self‐powered human motion monitoring.^[^ ^176^ ^]^ These TENGs are designed for wearable biomechanical energy‐harvesting and self‐powered human motion monitoring, with applications in biomedical fields and human‐machine systems.

Electrospinning technology has been used to prepare PAN/BaTiO3/MXene nanofiber membranes with high‐voltage electrical performance and photothermal conversion efficiency, combined with nickel copper conductive fabrics to construct piezoelectric frictional hybrid nanogenerators with superior electrical properties. These membranes, which combine the advantages of piezoelectric materials and nanogenerators, exhibit excellent photothermal properties and can be applied in photothermal therapy to relieve joint pain. Dan et al. developed a self‐promoting electroactive mineralization scaffold (sp‐EMS) by biomimetically assembling mineralized collagen fibers with silver ultrathin nanowires (Ag uNWs) to effectively address bacterial‐infected bone defects.^[^ ^177^ ^]^ The sp‐EMS consistently produces weak currents through gentle electrochemical reactions, stimulating the recruitment of BMSCs and promoting osteogenic differentiation and angiogenesis.^[^ ^177^ ^]^

As noted previously, TENGs show significant potential for the development of self‐powered devices. However, their sensitivity to moisture necessitates encapsulation within a protective layer, making them unsuitable as tissue‐engineered scaffolds that require direct contact with surrounding cells. Zhengwei et al. introduced friction electric scaffolds (TESs) made from polyglycerides (PGs), a biodegradable and hydrophobic elastomer.^[^ ^10^ ^]^ In multistage porous TESs, each hydrophobic and moisture‐resistant micropore efficiently serves as the working unit of TENGs, even in humid environments. The integration of a large number of micropores ensures the ability of TESs to perform electrotherapy in vivo without the need for encapsulation. Additionally, the initially hydrophobic TES is degraded by surface erosion and transformed into hydrophilic surfaces, contributing to its role as a tissue engineering scaffold.^[^ ^10^ ^]^ Notably, TESs inoculated with chondrocytes produced dense, mature cartilage after subcutaneous implantation in nude mice. Importantly, rabbits with osteochondral defects implanted with TESs exhibited good hyaluronic acid cartilage regeneration and complete cartilage healing. This study highlights TESs as a promising electronic biomedical device with potential for new in vivo applications.^[^ ^10^ ^]^ Shuangfei et al. utilized cell wall nanoengineering technology to construct a lightweight and ultra‐strong cellulose triboelectric material. This strategy allows for the design of a cell wall structure with multilevel pores interconnected at different scales and good structural stability. This material exhibits porous properties, good structural stability, and excellent output performance, making it a promising candidate for use in frictional electric sensors.^[^ ^178^ ^]^

In recent years, numerous scholars have conducted extensive research on the chemical structures and material properties of polymer materials in accordance with specific application scenarios. Researchers synthesized implantable polyzwitterionic hydrogel scaffolds using methacrylated sulfonated betaine (DMAPS) and acrylated gelatin (GelMA). The mechanical properties of the polyzwitterionic hydrogel were significantly enhanced, making it suitable for repairing bone tissue defects. Zwitterionic hydrogels can regulate fibronectin binding and metabolic reactions, mediate cell‐matrix interactions, recruit BMSCs, and significantly enhance osteoblastic differentiation, ultimately leading to significant improvements in osteogenesis (Figure 5d).^[^ ^179^ ^]^ These findings underscore the potential of zwitterionic hydrogels in bone tissue repair and their future clinical applications.

In recent years, there have been many new signs of progress in the application of electric materials in osteochondral repair, which is of great significance for exploring the improvement ideas of electric materials. Millisecond‐pulsed electrical stimulation could influence cellular activities and gene expression through ion channel activation, calcium oscillations, and chromatin accessibility. Epigenetic alterations such as histone acetylation modification can alter chromatin outcome and play an important role in ES induced cell behavior.^[^ ^180^ ^]^ A novel electrical stimulation system based on polypyrrole nanowires (ppyNWs) integrated within a conductive hydrogel matrix was developed to mimic the cell microenvironment and enhance bone marrow mesenchymal stem cell osteogenesis, demonstrating improved cell spreading, osteogenic markers, and bone‐related gene expression, with RNA‐seq analysis revealing connections to Notch, BMP/Smad, and calcium signal pathways.^[^ ^181^ ^]^ Liu D et al. engineered a piezoelectric‐conductive scaffold that harnesses joint movement to generate electrical potential, directing BMSCs toward chondrogenic differentiation in the upper layer and osteogenic differentiation in the lower layer, thus enhancing osteochondral repair. This piezoelectric‐conducive scaffold induced biphasic division of BMSCs to provide a promising platform for the repair of osteochondral defects.^[^ ^182^ ^]^ Silva JC et al. evaluated how the five distinct ES protocols impact the viability, proliferation, and osteogenic differentiation of human bone marrow‐derived mesenchymal stem ^−1^stromal cells, revealing that specific ES waveforms significantly influence tissue mineralization and osteogenic gene expression, underscoring the need for precise ES parameter optimization to enhance in vitro osteogenesis.^[^ ^183^ ^]^ The electrophysiological microenvironment, such as the fate of cells or bacteria, immune response, and micro‐biomechanics, plays an important role in bone regeneration, which is an important direction for the improvement of electrical materials for bone defect repair. Aoao Wang et al. were inspired by the complex physiological processes involved in the initial healing phase of fracture to develop the ZnO@PCL/PVDF piezoelectric nanofiber‐oriented scaffold. This nanofibrous scaffold can not only guide cell growth and promote osteogenic differentiation, but also produce piezoelectric signal output similar to the physiological electrical signal of normal bone tissue, thereby creating a favorable electrical microenvironment during bone defect repair. Importantly, the scaffold effectively inhibited local pathogen infection and excessive inflammatory response through the synergistic effect of ZnO nanoparticles, thereby creating a favorable regenerative microenvironment. This remote‐controlled composite piezoelectric scaffold provides a simple and effective solution for the treatment of bone defects.^[^ ^184^ ^]^ Therefore, as an external intervention, electrical stimulation is expected to accelerate the regeneration process of bone and cartilage by integrating electrical, biochemical, and mechanical signals. Future studies could further optimize electrical stimulation techniques and materials to improve therapeutic efficacy and safety. The applications of electrical biomaterials for bone regeneration are summarized, as shown in **Table** **2**.

**Table 2:** Applications of electrical biomaterials for bone regeneration.

Electrical biomaterials	Electric effect	Validation in vitro	Validation in vivo	Highlights	Ref.
GaN/AlGaN	SP: 34.07 and −74.04 mV	Markedly promoted the attachment, recruitment, spreading, and osteogenesis of BMSCs	Showed rapid and superior bone formation for the perfect simulation of the physiological potential of bone tissue	Sheded light on the application of III‐nitride materials in bone regeneration.	[214]
DMAPS/GelMA	Endogenous electric field of controllable electrical stimulation	Exhibited high stereo‐affinity of fibronectin III7‐10, accelerated MSC recruitment and osteogenesis, inhibited inflammatory response	Significant improvement in osteogenic ability and better repair of bone defects	Zwitterionic gel provides excellent endogenous electric field and 3D for bone regenerationchemical microenvironment, and plays an important role in bone tissue repair and future clinical transformation applications	[179]
Flexible hybrid tribo/piezoelectric nanogenerator (HTP‐NG) and hydrogel	Provided biphasic electric pulses	Enhance multiple osteogenesis‐related biological processes, including calcium ion import and osteogenic differentiation	Bone defect was reversed by electrical stimulation therapy with BD‐ES and subsequent bone mineralization, and the femur completely healed within 6 weeks	Utilized the body's own rehabilitation exercise to provide stable and controllable electrical stimulation without the need for battery power and circuit modulation, saving the additional volume and mass required for power and circuits, and improving comfort	[5, 12]
Consisting of a triboelectric nanogenerator (TENG) and a flexible interdigitated electrode	self‐powered electrical stimulator	Promoted multiple biological processes such as adhesion, proliferation, and differentiation of osteoblasts	Movements successfully drived TENG to output electrical signals, stimulating bone formation and regeneration	Used TENG to convert the mechanical energy of human activities into electrical pulses, stimulating osteoblasts and enhancing their activity, achieving wearable and self driven electrical stimulation for bone formation and regeneration.	[172]
The TENG structure with an ion‐impregnated intermediate layer inserted to induce an ionic EDL (iEDL‐TENG)	current density (10–92 mA m^−2^) and a 13‐fold increase in power density (2–26 W m^−2^)			The ion‐containing electrolyte polymer was used as the interlayer to effectively mitigate charge attenuation and maximize TENG efficiency	[215]
PpyNWs/hydrogel	electrical stimulation system was determined to be 2 V	Significantly promoted cell spreading, osteogenic makers, and bone‐related gene expression of stem cells		Exogenous electrical stimulation expedited the osteogenic differentiation and facilitated the healing process of bone defects.	[181]
dECM/ Gel‐PC	Provided biphasic electric pulses	Directing BMSCs toward chondrogenic differentiation in the upper layer and osteogenic differentiation in the lower layer, thus enhancing osteochondral repair.	Effective repair of osteochondral defect	This piezoelectric‐conducive scaffold induced biphasic division of BMSCs to provide a promising platform for the repair of osteochondral defects.	[182]
ZnO@PCL/PVD	Ultrasound‐driven piezoelectric stimulation enhanced the proliferation, migration, and osteogenic differentiation of MC3T3‐E1 cells, inhibited bacterial colonization, and reduced inflammatory responses	Inhibit bacterial colonization, and reduce inflammatory response	Effectively promoting bone repair by accurately replicating the native electrical microenvironment and precisely regulating the temporal and spatial abnormalities during initial bone healin	The ZnO@PCL/PVDF composite exhibits multifunctional properties, including antibacterial, immunomodulatory, and osteogenic effects, making it a straightforward and efficient solution for addressing bone defects	[184]

### 3.2 Electric Stimulation for Cartilage Regeneration

The piezoelectric properties of articular cartilage arising from the presence of collagen molecules suggest that the incorporation of piezoelectric materials could be beneficial in engineered cartilage constructs. Previous studies have demonstrated the influence of mechanical stress and electromagnetic stimuli on chondrocyte behavior. However, the potential role of piezoelectric materials in promoting cartilage regeneration remains relatively unexplored. Mitani et al. used a piezoelectric PVDF membrane to culture multi‐layered chondrocyte sheets. The cells adhered well to the PVDF membrane, with increased gene expression of fibronectin and integrin α10, and maintained a phenotype similar to regular chondrocytes, with increased SOX9, Col X, and Col VII expression.^[^ ^185^ ^]^ Recently, Jacob et al. developed piezoelectric BT nanoparticle‐reinforced PHBV nanohybrid electrospun scaffolds that enhanced mechanical strength and stimulated cartilage regeneration without additional chemical factors. These findings indicate that piezoelectric scaffolds promote cartilage regeneration and can be used as bilayer scaffolds for osteochondral healing, providing simultaneous stimulation of cartilage and bone‐forming cells.^[^ ^167^ ^]^

**Figure 6:**
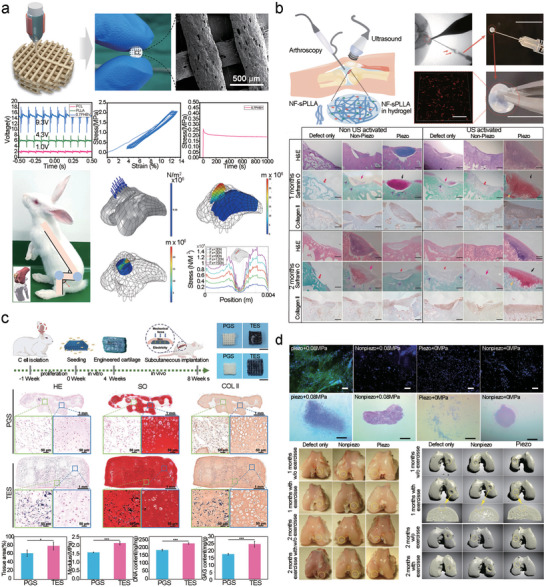
The applications of electroactive biological materials for cartilage regeneration. a) Macrophotography, electromechanical response, cyclic compression, and scanning electron microscopy (SEM) images of piezoelectric 3D scaffolds fabricated with 0.7PHBV.^[^ ^187^ ^]^ Copyright 2024, Cell. b) Piezo PLLA hydrogels produce better articular cartilage regeneration compared with sham and non‐piezo PLLA hydrogels by H&E, Safranin O/fast green, and collagen II staining.^[^ ^190^ ^]^ Copyright 2023, Springer Nature. c) Triboelectric scaffolds (TESs) promote cartilage regeneration compared with Poly (glycerol sebacate) (PGS).^[^ ^10^ ^]^ Copyright 2024, Wiley‐VCH. d) Piezoelectric scaffold promotes ADSC chondrogenesis under, and exercise‐induced piezoelectric stimulation enhances bone and cartilage regeneration.^[^ ^5^ ^]^ Copyright 2024, AAAS.

You et al. proposed a friction electric stent without encapsulation using a polyglycerol ester (PGS) that successfully combined electrotherapy with traditional tissue‐engineering scaffolds to form a single bioabsorbable device (**Figure** **6**c).^[^ ^10^ ^]^ This scaffold performs excellently in promoting cartilage regeneration, generating pulsed ES similar to natural electrical signals in the body, and effectively promoting the regeneration and repair of the cartilage and subchondral bone.^[^ ^10^ ^]^ Ouyang et al. reported a smart “tissue battery” based on friction nanogenerators for cartilage repair therapy and real‐time monitoring of the repair process. They Utilized self‐generated microcurrents to stimulate rapid cartilage repair and achieved real‐time visualization of the “black box” during the cartilage repair process, achieving the goal of “inducing tissue regeneration – real‐time monitoring of repair” integration.^[^ ^186^ ^]^ Therefore, this frictional electric stent shows great potential for clinical translation and can be used to repair cartilage and other tissues.

Researchers have demonstrated that biodegradable PLLA nanofiber scaffolds can function as battery‐free electrical stimulators to facilitate cartilage formation and regeneration under applied forces or joint loads. They created a three‐layer biodegradable piezoelectric PLLA nanofiber scaffold that activates the calcium signaling pathway under external force or joint load, enhances TGF‐β secretion, and promotes extracellular protein adsorption, cell migration, and recruitment, thereby promoting cartilage formation and regeneration in vitro and in vivo (Figure 6a).^[^ ^187^, ^188^, ^189^ ^]^ Following the administration of piezoelectric scaffolds and exercise therapy, rabbits with extensive bone and cartilage defects exhibited transparent cartilage regeneration within 1–2 months (2–3 months post‐surgery, including a one‐month recovery period prior to exercise). Subsequently, complete cartilage healing was observed along with abundant levels of chondrocytes and Col II. Conversely, rabbits treated with nonpiezoelectric scaffolds and exercise therapy exhibited unfilled defects and limited healing. Consequently, the combination of biodegradable piezoelectric tissue scaffolds with controlled mechanical activation (via physical exercise) holds promise for osteoarthritis treatment and regeneration of other injured tissues (Figure 6b).^[^ ^190^ ^]^ These findings demonstrate that under controlled mechanical stimulation, piezoelectric scaffolds can generate surface charges conducive to cartilage growth, thereby enhancing the in vitro differentiation of stem cells and promoting cartilage regeneration (Figure 6d).^[^ ^5^ ^]^ The applications of electrical biomaterials for cartilage regeneration are summarized, as shown in **Table** **3**.

**Table 3:** Applications of electrical biomaterials for cartilage regeneration.

Electrical biomaterials	Electric effect	Validation in vitro	Validation in vivo	Highlights	Ref.
CA‐CS‐HA+/PCL/PDMS/PCL‐FA+PCL‐PLGA‐HA	Self‐generated microcurrents	The tissue‐battery generate electricity to stimulate chondrocyte proliferation	Accelerated cartilage repair; visualized the state of cartilage repair by sending electrical signals to feedback structural changes caused by material degradation and cell proliferation	Utilized self‐generated microcurrents to stimulate rapid cartilage repair and achieving real‐time visualization of the "black box" during the cartilage repair process, achieving the goal of "inducing tissue regeneration ‐ real‐time monitoring of repair" integration.	[186]
PLLA/collagen	A battery‐less electrical stimulator converted pressure into electrical current to provide endogenous electric field	Promoted extracellular protein adsorption, facilitated cell migration or recruitment, induced endogenous TGF‐b via calcium signaling pathway, and improved chondrogenesis inflammatory response	Completely healed cartilage with abundant chondrocytes and type II collagen after 1 to 2 months of exercise	Applied force or joint load could act as a battery‐less electrical stimulator and generated a controllable piezoelectric charge to promote chondrogenesis and cartilage regeneration.for bone regenerationchemical microenvironment, and plays an important role in bone tissue repair and future clinical transformation applications	[5]
PGS/PEDOT: PSS	TESs with 5 wt% PPGS can maintain stable electrical output after 1000 cycles of compression. TESs can also generate electrical energy similar to natural electrical signals in the body, with a voltage output of approximately 0.2 V, which is comparable to natural electrical signals in the body		Electrical stimulation has a significant promoting effect on the growth of chondrocytes and the maturation of cartilage tissue	The dynamic hydrophilic hydrophobic transition results in the formation of hydrophilicity on the surface during degradation to promote tissue regeneration, while the internal micropores maintain hydrophobicity for frictional electrical therapy. The unique design makes it possible to directly apply friction nanogenerators in vivo without packaging	[10]
PLLA/collagen	The output voltage of NF sPLLA dried hydrogel scaffold is ∼33.7 mV peak‐to‐peak,	Piezoelectricity induced chondrogenesis by recruiting the stem cells and stimulating the cells to secrete endogenous TGF‐β1.	Induces cartilage healing, subchondral bone formation, and alleviated osteoarthritis	This piezoelectric hydrogel is not only useful for cartilage healing but also potentially applicable to osteoarthritis treatment	[190]
PHBV/ PLLA	possessed a piezoelectric coefficient of 2.0–2.5 times greater than PLLA		Excellent cartilage regeneration was induced in the 0.7PHBV	Self‐powered bio‐piezoelectric materials for remodeling tissues in stressed sites through piezoresponse	[187]
					

### 3.3 Clinical Translatability of Electrical Biomaterials

Electroactive biomaterials are progressively demonstrating immense potential in the clinical application of bone and cartilage regeneration. These materials exhibit a high degree of design flexibility, enabling diverse processing needs such as injectability, minimal invasiveness, and biodegradability, which facilitate their practical and safe application in clinical settings. More importantly, electroactive biomaterials can seamlessly synergize with various bioactive factors or established scaffolds, jointly acting on damaged bone and cartilage tissues to promote cell proliferation, differentiation, and migration, thereby accelerating tissue repair and regeneration processes.^[^ ^167^ ^]^ In multiple disease models, including fracture healing, bone defect repair, cartilage injury repair, and neural regeneration, electroactive biomaterials have achieved significant therapeutic effects, offering patients new treatment options and hope.^[^ ^191^, ^192^ ^]^ However, despite the substantial potential of electroactive biomaterials in bone and cartilage regeneration, numerous challenges and difficulties remain in their clinical translation. Firstly, the long‐term biosafety of these materials cannot be overlooked. Although some studies have reported the promotive effects of electroactive biomaterials on bone and cartilage regeneration, the understanding of their long‐term efficacy and mechanisms is still relatively limited. In particular, further in‐depth research is required into the biocompatibility and metabolic pathways of the degradation products of biodegradable polymer materials, as well as those of nanoparticle materials.^[^ ^166^ ^]^ Secondly, the stability of material performance in complex in vivo environments poses an urgent problem to be solved. Ensuring the materials' continuous and stable electrical signal output in vivo and effectively monitoring their performance changes is a major challenge in current research. This not only necessitates more innovations and optimizations in material design but also requires the establishment of stricter and more standardized monitoring systems in clinical trials. Moreover, achieving a balanced performance in electrical, mechanical properties, lubricity, and micro‐morphology of electroactive materials demands further resolution. Such balance is crucial to fulfilling the diverse needs of clinical implants, thereby enhancing therapeutic outcomes and patient rehabilitation experiences. Therefore, future research necessitates more efforts and explorations in material design, performance optimization, and clinical trials to promote the further development of electroactive biomaterials in clinical translation.

## 4 Biological Response to Electric Stimulation

### 4.1 Electric Stimulation in Tissue Regeneration

ES, an important biophysical regulatory factor and non‐pharmacological intervention in clinical practice, has received widespread attention owing to its ability to regulate cell activity and enhance tissue repair.^[^ ^193^, ^194^ ^]^ However, the detailed mechanism by which electrical materials promote bone repair is still unclear. Several studies have shown that human bones exhibit piezoelectric properties.^[^ ^1^ ^]^ When bones undergo deformation under force, charges are generated inside them, particularly when subjected to periodic deformation, which can generate a regular electron flow. This piezoelectric signal can affect the differentiation of bone cells and regulate their functions. Piezoelectricity is the ability of a material to generate an electric field when subjected to mechanical stress. The creation of surface charges results from the misalignment of internal dipoles owing to external mechanical forces.^[^ ^195^ ^]^ Given the inherent piezoelectric properties of natural bone tissue, there is growing interest in electroactive piezoelectric materials for enhancing the effectiveness of bone repair and regeneration therapies. These materials can harness the natural deformation that occurs in the bone during movement as a platform for mechanical‐to‐electrical conversion, producing immediate bioelectric stimulation and transforming it into electrophysiological signals that mimic physiological activities. When implanted, these materials generate electrical signals through their surface charges under mechanical stress, which promotes protein adhesion, modifies the membrane potential, regulates voltage‐gated calcium channels, and ultimately influences various cellular behaviors. Additionally, piezoelectric materials can encourage macrophage polarization toward the M2 phenotype, aiding in the regulation of local inflammatory responses and fostering an optimal microenvironment for tissue regeneration. Therefore, piezoelectric materials are promising electroactive biomaterials for addressing bone defects.^[^ ^196^ ^]^

### 4.2 Electric Stimulation‐Induced Cell Response

**Figure 7:**
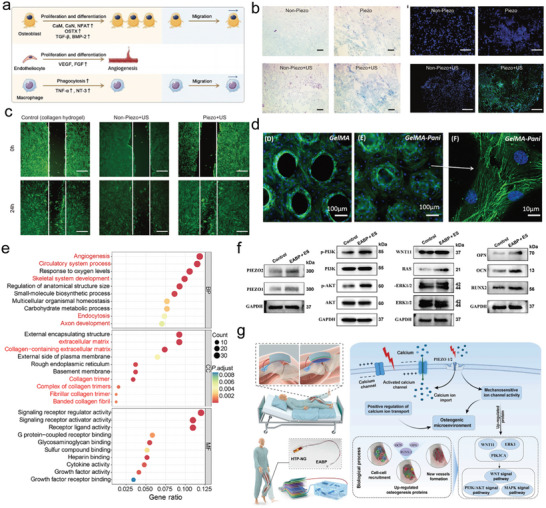
Exploration of electric stimulation‐induced molecular and cell responses. a) Electrical stimulation accelerates proliferation and differentiation of osteoblasts, promotes vascularization, and improves macrophage phagocytosis in the process of bone healing.^[^ ^199^ ^]^ Copyright 2024, Wiley‐VCH. b∼c) Scratch test exhibits the migration of stem cell by treated with control, Piezo, and collagen hydrogel (Scale bars: 500 µm).^[^ ^190^ ^]^ Copyright 2023, Springer Nature. d) Bright‐field images and fluorescence images of GelMA and GelMA‐Pani by digital projection micro‐GelMA‐Pani produced by stereolithography.^[^ ^216^ ^]^ Copyright 2016, Elsevier. e) GO functional enrichment analysis including cellular component, biological process, and molecular function.^[^ ^12^ ^]^ Copyright 2024, AAAS. f) The gene expression patterns in cells after electric stimulations.^[^ ^12^ ^]^ g) Schematic of the battery‐free Bd‐eS for patients performing active and the possible mechanism for bone and cartilage repair. Copyright 2024, AAAS.^[^ ^12^ ^]^

Because of the intricate interplay between collagen and bones, bones exhibit piezoelectricity, with an internal potential ranging from −60 to −100 mV under normal physiological load. Electrical activity within the bone microenvironment plays a pivotal role in the regulation of bone homeostasis and healing. This review comprehensively examines the impact of ES on various cellular behaviors, including proliferation, adhesion, alignment, migration, and differentiation.^[^ ^197^ ^]^ Furthermore, a comprehensive summary of the applications of electrically active biomaterials (EABMs) in bone tissue repair is provided from five perspectives: proliferation enhancement, osteoblast migration facilitation, osteogenesis promotion, and adhesion improvement (**Figure** **7**a,b). Additionally, this review provides a thorough analysis and synthesis of plausible mechanisms based on existing research findings. By harnessing the influence of external electric fields on the ability of conductive materials to generate localized electrical signals in situ, precise regulation of cell behavior and effective bone regeneration can be achieved.^[^ ^198^ ^]^

The response of cells to ES is complex and not fully understood. The cell membrane, encompassing negatively charged chemical groups, such as carboxylates and phosphates, serves as the primary interface with the surrounding environment. Electrical stimulation plays a crucial role in enhancing bone regeneration through various biological mechanisms. By applying electrical currents to the site of bone injury, the proliferation and differentiation of osteoblasts are significantly accelerated, resulting in expedited and more robust regeneration of bone tissue. Furthermore, this stimulation promotes vascularization, facilitating the development of new blood vessels that enhance blood supply to the healing area. Additionally, electrical stimulation augments the phagocytosis of macrophage, which contributes to maintaining a clean and favorable environment for bone regeneration, ultimately supporting a more efficient process (Figure 7a).^[^ ^199^ ^]^ Soluble ions within ion pumps, transporters, and channels regulate cellular activity by influencing membrane proteins that are responsive to external stimuli such as electricity. Specific regulatory membrane proteins and enzymes can perceive and employ electric fields for regulatory purposes. Once a cell adheres to a surface, it employs ion channels and receptors in the membrane to assess its surroundings before establishing focal adhesions through integrin binding with extracellular matrix (ECM) ligands.^[^ ^200^ ^]^

Electrical stimuli can be applied through either the medium or the substrate. ES using conductive polymers (CPs), such as PPy and PANI, offers indirect methods for ES.^[^ ^201^ ^]^ CPs have demonstrated a positive impact on cell proliferation and extension when used as a substrate, even without the addition of an electrical current, compared to samples lacking a CP‐based substrate. For example, applying a direct surface current of 10 µA significantly increased fibronectin (Fn) adsorption, particularly during early exposure to high Fn concentrations. Neurite growth in PC‐12 cells cultured on these stimulated films was up to 50% longer than that on unstimulated films. Schmidt et al. conducted an experiment in which an electrical field of 100 mV mm^−1^ was applied to a PPy‐coated electrospun scaffold, resulting in a significant increase in both the number and length of neurite‐bearing cells. These findings demonstrate the potential applications of ES with electroactive polymers for tissue regeneration.^[^ ^202^ ^]^

Piezoelectric polymers have been utilized to replicate the electrical conditions found in the bone, resulting in the stimulation of osteoblast migration (Figure 7c,d). Additionally, bioglass nanofibers have been used to release various mineral ions, including Ca^2+^ and P^4+^ ions. Further investigation revealed that PVFT‐BGM facilitated osteogenesis by activating calcium‐sensitive receptors present in osteoblasts and influencing subsequent signaling pathways. The scaffold demonstrated its capability to enhance the growth, proliferation, and differentiation of bone marrow stem cells while also promoting the development of periosteum‐like tissue and facilitating bone regeneration.^[^ ^203^ ^]^

Research has been conducted on the use of Poly(3,4‐ethylenedioxythiophene) (PEDOT) substrates to control electrical signals in ECM proteins, such as Fn, that regulate cellular interactions. The aim of these studies was to explore the potential of electrically controlling cellular redox properties and their applications in cell culture experiments.^[^ ^204^ ^]^ By applying ES, changes in the conformational states of ECM proteins can be induced, leading to alterations in cellular responses such as cell adhesion. Oxidized PEDOT coated with Fn promotes specific protein conformations that facilitate cell attachment and proliferation. Recent advancements have demonstrated that heparin‐doped oxidized PEDOT effectively releases the fibroblast growth factors anchored to its surface, thereby promoting stem cell differentiation through precise electrochemical switches. This highlights the correlation between electrochemical modulation and the accurate sequential regulation of stem cell states.

The ongoing discussion about whether a positively or negatively charged surface is more effective in promoting osteogenic differentiation remains unresolved. Because of the wurtzite structures of GaN and AlGaN, which exhibit opposing spontaneous polarizations, it is feasible to manipulate the heterostructures of these materials by varying the direction and magnitude of their spontaneous and piezoelectric polarizations. This manipulation ultimately affects surface charge. Zhang et al. successfully developed two piezoelectric heterostructured materials with contrasting surface charges: negatively charged Ga‐GaN/AlGaN and positively charged N‐GaN/AlGaN. The experimental results demonstrated that negatively charged Ga‐GaN/AlGaN significantly improved the attachment, osteogenic differentiation, diffusion, and recruitment of bone mesenchymal stem cells.^[^ ^2^ ^]^ Furthermore, these results indicate that bone morphogenetic protein‐6 may act as an electrosensitive protein during early‐stage osteogenic differentiation. However, additional studies are needed to fully elucidate the underlying mechanisms.

### 4.3 Electric Stimulation‐Induced Molecular Response

ES induced by piezoelectricity and direct mechanical stimulation can facilitate the healing of damaged tissues. These mechanisms can be explained by two separate components. According to the first component, when functional loads are applied, the piezoelectric scaffolds generate an electrical potential. This type of ES activates pathways involving voltage‐gated Ca^2+^ channels and stretch‐activated calcium channels, leading to increased intracellular Ca^2+^.^[^ ^205^ ^]^ Additionally, a local electrical field can alter the configuration of membrane receptors and open receptor channels, allowing intracellular Ca^2+^ ions from the endoplasmic reticulum to enter. Elevated levels of Ca^2+^ within cells activate calcium‐modulated proteins and a calcium‐dependent protein phosphatase, calcineurin. Calcineurin interacts with phosphorylated nuclear factors of activated cells (NF‐AT‐PO_4_), converting them into dephosphorylated nuclear factors of activated cells (NF‐AT). These dephosphorylated NF‐AT then move into the nucleus, where they bind to other proteins and initiate gene transcription. Gene transcription promotes the synthesis of TGF‐β and BMP‐2, thereby regulating cell metabolism and ECM synthesis. In the second component, mechanical stimulation directly activates mechanoreceptors, such as integrins. Once activated, integrins transport protein kinase C (PKC) to the cell membrane, triggering MAPK pathways for transmembrane signaling. Consequently, extracellular mechanical signals are transmitted to actin.^[^ ^91^, ^206^ ^]^ Receptor‐associated protein RACK1, located within the cell, forms a complex with integrins and binds to the PKC domain. Consequently, the interaction between RACK1 and PKC‐integrin plays a crucial role in the transmission of signals across the cell membrane. These signaling cascades then proceed toward the nucleus, where they interact with transcription factors sensitive to mechanical stimuli, ultimately leading to gene transcription. It can be inferred that this signaling cascade primarily consists of G protein‐coupled calcium channels. The regulation of adenosine triphosphate (ATP) and reactive oxygen species (ROS) levels is predominantly governed by the mitogen‐activated protein kinase (MAPK) and Phosphatidylinositol 3‐kinase/Protein Kinase‌ (PI3K/AKT) pathways (Figures 2h and 7e–g).^[^ ^84^ ^]^

The piezoelectric nature of bone collagen leads to the generation of both negative and positive potentials when subjected to mechanical deformation, with compression resulting in a negative potential, and tension resulting in a positive potential. Bones possess an inherent zeta potential of −5 mV, which is further enhanced by the development of negative charges from compressed bone collagen.^[^ ^87^ ^]^ This stress‐induced electrical potential improves electroosmosis, reduces hydraulic permeability, and increases bone stiffness. Articular cartilage is a load‐bearing tissue composed mainly of collagen molecules (type II, VI, IX, X, and XI), proteoglycans, non‐collagenous proteins, and fluids. It also exhibits piezoelectric properties similar to bone owing to its collagen composition. Therefore, exploring the use of piezoelectric materials for engineered cartilage constructs is promising as the cartilage itself consists of piezoelectric components.^[^ ^207^ ^]^ Although studies have been conducted on the effects of mechanical stress (shear, compressive, or hydrostatic) and electromagnetic stimuli on chondrocyte behavior in cartilage regeneration, little attention has been paid to the direct relationship between piezoelectricity and cartilage regeneration.^[^ ^208^ ^]^

### 4.4 Potential Mechanisms of Electric Stimulation‐Induced Osteochondral Repair

ES is a promising external intervention with the potential to accelerate the healing of bone defects. Natural electric fields within our bodies play a vital role in regulating bone health and regeneration, making them important biophysical factors. Electroactive materials found in bone tissue generate bioelectric signals that influence various aspects of bone healing, such as cell adhesion, proliferation, arrangement, migration, and differentiation. There are two main types of EABMs based on their source of electrical signals: conductive biomaterials capable of transmitting electrical signals, and piezoelectric biomaterials that can generate electrical signals themselves. This review highlights how these materials significantly advance osteogenesis, chondrogenesis, angiogenesis, and possess antibacterial properties during bone healing.^[^ ^209^ ^]^

EnEFs play a crucial role in biological processes such as embryogenesis, physiological activity, wound healing, and tissue remodeling, with the bone's EnEF originating from the piezoelectric effect of the collagen matrix. Hydrogels such as methacryloyl gelatin (GelMA)‐PANi are ideal for 3D scaffolds and can be prepared using enhanced interfacial polymerization. Electroactive fiber membranes have been fabricated by self‐assembling and chemically reducing graphene oxide on PVC/PLGA nanofibers. Coating 316 L stainless steel alloy (316L‐SA) with AgNPs/PLGA induces electron flow, creating a positive surface potential for local osteogenesis and bone fusion. To prevent BT nanoparticle aggregation, DOPA modification is performed before mixing with PVDF to form a piezoelectric DOPA@BT thin film. After corona polarization, this film maintains a surface potential of −76.8 mV and retains over half of its charge in vivo for 12 weeks (Figure 3b). Saos‐2 osteoblast migration onto the ZnO nanosheets generates an electric field that enhances cell activity without the adverse effects of aggregation or other factors.^[^ ^210^ ^]^

The utilization of a 3D flexible nanofiber P (VDF TrFE) scaffold is a significant milestone in evaluating the differentiation of BMSCs into cartilage and bone cells. In the case of skull defects, analysis through the 3D reconstruction of blood vessels revealed that the presence of ES had a notable impact on promoting both bone maturation and angiogenesis.^[^ ^210^ ^]^ The incorporation of Na0.5K0.5NbO_3_ into positively or negatively polarized composite materials contributes to their exceptional antibacterial properties by potentially increasing superoxide production, particularly on positively charged surfaces. Furthermore, the application of a polarized electric field can disrupt Fe‐S clusters found in proteins, leading to Fe^2+^ reacting with H_2_O_2_ to generate free radicals that damage bacterial membranes. By harnessing its electrical activity, this composite scaffold facilitates the controlled release of the human bone morphogenetic protein‐4 (hBMP‐4) gene and enhances osteogenic differentiation.^[^ ^211^ ^]^

Surfaces with positive charges tend to attract negatively charged ions such as Cl^–^ or HPO^4–^, which can hinder cell adhesion. In physiological conditions, N‐GaN/AlGaN and Ga‐GaN/AlGaN materials have surface potentials of −34.07 and −74.07 mV, respectively. Dexamethasone, which negatively charges osteogenic induction media, can boost the differentiation of MSCs into bone cells. An electric field can be generated between a BiFeO3 film, having a +75 mV surface potential, and a negatively charged area within a bone defect, which can help in binding Fn molecules and enhance in vivo bone integration by promoting cell diffusion, adhesion, and migration.^[^ ^212^ ^]^

EnEFs, an essential element in living organisms, play crucial roles in the regulation of growth, development, and signal transmission. Unlike neurons and cardiomyocytes, bone cells lack the ability to generate action potentials; however, they are influenced by the piezoelectric properties of bone's collagenous structure. This structure creates an internal potential of −60 to −100 mV under physiological load, which is crucial for bone homeostasis and healing. This review examined the effects of ES on cell behavior including proliferation, adhesion, alignment, migration, and differentiation. It also examined the role of ES in bone tissue repair, emphasizing its effects on osteogenesis, chondrogenesis, and angiogenesis, as well as its antibacterial properties and drug delivery efficacy. This study also analyzed potential mechanisms, suggesting that alterations in calcium channels, G‐proteins, ATP, and ROS play crucial roles in signaling pathways regulated by MAPK and PI3K/Akt.^[^ ^213^ ^]^

**Figure 8:**
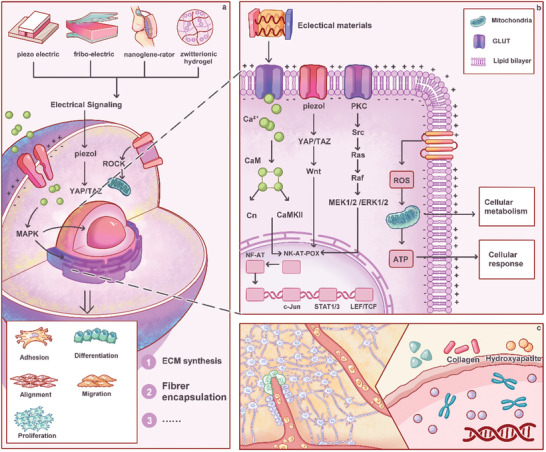
Illustration shows the ECM of bone and cartilage repair as well as the underlying mechanisms.

The piezoelectric characteristics of bones have been recognized as crucial for maintaining the physiological functionality of bones. Bones possess the ability to autonomously generate internal electrical signals that arise from polarization resulting from the displacement of collagen fibrils when subjected to shear force. These subtle electrical signals play a pivotal role in directing stem cell differentiation and tissue regeneration, enhancing calcium influx, triggering the Ca^2+^ signaling pathway, boosting osteogenic differentiation of stem cells, stimulating cellular metabolism, and influencing membrane‐related cellular behavior. Furthermore, electric stimulation has been demonstrated to facilitate the attachment and proliferation of osteoblasts while impeding bacterial activity (**Figure** **8**).

## 5 Conclusion and Outlook

EnEFs are integral to biological systems and play crucial roles in growth regulation and signal transduction. Unlike neurons and cardiomyocytes, bone cells are incapable of generating spontaneous action potential generation. However, the piezoelectric properties of bone, attributed to its collagen content, produce an electric field of 60–100 mV under physiological loads, which is essential for bone regeneration. We investigated the regulatory effects of electrical stimulation at the molecular, cellular, and tissue levels, such as cell proliferation, differentiation, migration, adhesion, upregulation or downregulation of related pathway activity, and tissue shaping and remodeling. The underlying mechanisms, potentially involving calcium channels, G‐proteins, ATP, and ROS, which are regulated by the MAPK and PI3K‐Akt pathways, were discussed. This review highlights the need for rigorous application of ES in vitro and advocates the use of clinically approved bone stimulators as electrical signal sources.

Clinical implications: direct current (DC) stimulation, with an optimal intensity range of 5–20 µA, presents a risk of osteonecrosis at higher currents. The Faraday reaction induced by ES enhances osteoblast activity and promotes angiogenesis. Despite the improved safety of noninvasive stimulators, their efficacy is time‐dependent, potentially affecting patient adherence to treatment protocols. The specifications for safe operation of continuous current (CC) and integrated circuit (IC) equipment are detailed, emphasizing the need for self‐powered ES devices owing to their safety and potential for improved patient compliance.

Material considerations: Piezoelectric materials offer safer alternatives to traditional ES devices by eliminating the need for implanted electrodes and wires. However, the contradictory findings regarding the influence of surface charge on cellular processes require further investigation. This review also examined the challenges of integrating piezoelectric materials with TENGs and the limitations of photovoltaic cells in bone repair applications. The development of implantable EABMs has been proposed as a solution to address the high cost and lengthy treatment cycles associated with ES therapy, with a focus on mimicking the bone structure and optimizing mechanical properties, degradation rates, and electrical signal delivery.

Future directions: Electrical stimulation is acknowledged for its capacity to promote cell proliferation and directed differentiation, particularly in the activation of osteoblasts and chondrocytes. Studies have demonstrated that an electric field can influence the polarization state of cellular membranes, enhance calcium ion channel activity, and increase intracellular calcium ion levels—factors that are essential for the differentiation of specific cells within bone and cartilage tissues. When mechanical pressure is applied to nanogenerators and piezoelectric materials, they generate microcurrents that directly stimulate stem cells to differentiate into osteoblasts or chondrocytes, thereby aiding in the regeneration of bone and cartilage tissues. Moreover, electrical stimulation can trigger directed cell migration—a process known as “electrotaxis.” This phenomenon is crucial for rapid tissue repair processes, especially in healing bone and cartilage; under an electric field's influence, cells move toward damaged regions, thus facilitating regeneration in these affected areas. Additionally, triboelectric scaffolds can accelerate tissue repair by promoting surrounding cell migration toward injury sites through microcurrents generated by triboelectric effects. Electrical stimulation also modulates both the synthesis and degradation of extracellular matrix (ECM), creating optimal conditions for regeneration. The electric field stimulates collagen production along with cartilage matrix proteins such as chondroitin sulfate while enhancing matrix metalloproteinase (MMP) activity—thereby effectively maintaining a dynamic balance within the ECM. Zwitterionic hydrogels act as electroactive biomaterials that not only demonstrate excellent electrical conductivity but also improve interactions between cells and their matrices when subjected to external electric fields; this enhances ECM distribution while supporting bone and cartilage tissue regeneration. During regenerative processes involving bone and cartilage, inflammatory responses may adversely affect tissue repair outcomes. Research indicates that moderate electrical stimulation can suppress pro‐inflammatory cytokine release while promoting anti‐inflammatory factor expression—thus fostering a more favorable environment for early‐stage regeneration. For instance, microcurrents produced by nanogenerators have been shown to inhibit macrophage activation while decreasing inflammatory factor production—ultimately leading to enhanced tissue regeneration.

Future research in the domain of bone and cartilage regeneration should build on recent progress in electro‐biomaterials by concentrating on the following key areas: 1) Examine how variations in electric field intensity, frequency, and duration affect bone and cartilage cells to determine the most effective combinations of electrical stimulation parameters that enhance regenerative results. 2) Combine specific growth factors (such as bone morphogenetic protein BMP and transforming growth factor TGF) with electrical stimulation to investigate their combined effects on the proliferation and differentiation of bone and cartilage cells. 3) Create electro‐biomaterials designed for responsive and controllable release characteristics, enabling real‐time adjustments to external stimuli and material reactions. 4) Utilize advanced methodologies like transcriptomics and proteomics to thoroughly investigate the regulatory mechanisms through which electrical stimulation impacts cell signaling pathways, gene expression, and protein synthesis, thereby clarifying the biological principles behind electrical stimulation's role in bone and cartilage regeneration. The use of electro‐biomaterials offers an innovative strategy for improving bone and cartilage repair processes. Incorporating electrical stimulation technology into this area of study will provide a solid theoretical foundation along with practical support for further advancements.

This review concludes with a call for the development of intelligent bone‐implantable scaffolds that integrate osteogenic and chondrogenic capabilities. It highlights the promise of electroactive biomaterials in improving bone and cartilage repair, while also recognizing the need to address current technological limitations.

## Conflict of Interest

The authors declare no conflict of interest.

## References

[1] F. Barbosa , F. C. Ferreira , J. C. Silva , Int. J. Mol. Sci. 2022, 2907, 23. 35328328 10.3390/ijms23062907 PMC8952277

[2] Y. Du , J. L. Guo , J. Wang , A. G. Mikos , S. Zhang , Biomaterials 2019, 218, 119334. 31306826 10.1016/j.biomaterials.2019.119334 PMC6663598

[3] Y. Bai , Z. Wang , X. He , Y. Zhu , X. Xu , H. Yang , G. Mei , S. Chen , B. Ma , R. Zhu in Bone Tissue Engineering. Small methods 2024, 8, 2301283. 10.1002/smtd.202301283 38509851

[4] D. T. Dixon , C. T. Gomillion , Journal of functional biomaterials 2021, 1, 13. 10.3390/jfb13010001 PMC8788550 35076518

[5] Y. Liu , G. Dzidotor , T. T. Le , T. Vinikoor , K. Morgan , E. J. Curry , R. Das , A. McClinton , E. Eisenberg , L. N. Apuzzo , K. T. M. Tran , P. Prasad , T. J. Flanagan , S. W. Lee , H. M. Kan , M. T. Chorsi , K. W. H. Lo , C. T. Laurencin , T. D. Nguyen , Sci. Transl. Med. 2022, 14, abi7282. 10.1126/scitranslmed.abi7282 35020409

[6] M. Alrwaily , M. Schneider , G. Sowa , M. Timko , S. L. Whitney , A. Delitto , Brazilian journal of physical therapy 2019, 23, 506. 30482602 10.1016/j.bjpt.2018.10.003 PMC6849075

[7] W. Zhang , Y. Luo , J. Xu , C. Guo , J. Shi , L. Li , X. Sun , Q. Kong , The Possible Role of Electrical Stimulation in Osteoporosis, A Narrative Review Medicina (Kaunas, Lithuania) 2023, 59, 121. 36676745 10.3390/medicina59010121 PMC9861581

[8] L. Juckett , T. M. Saffari , B. Ormseth , J. L. Senger , A. M. Moore , Biomolecules 2022, 12, 1856. 36551285 10.3390/biom12121856 PMC9775635

[9] H. Feng , C. Zhao , P. Tan , R. Liu , X. Chen , Z. Li , Nanogenerator for Biomedical Applications. Advanced healthcare materials 2018, 7, 1701298. 10.1002/adhm.201701298 29388350

[10] B. Luo , S. Wang , X. Song , S. Chen , Q. Qi , W. Chen , X. Deng , Y. Ni , C. Chu , G. Zhou , X. Qin , D. Lei , Z. You , Advanced materials (Deerfield Beach, Fla) 2024, 36, 2401009. 10.1002/adma.202401009 38548296

[11] X. Wang , J. Song , F. Zhang , C. He , Z. Hu , Z. Wang , Advanced materials (Deerfield Beach, Fla) 2010, 22, 2155. 20564252 10.1002/adma.200903442

[12] T. Wang , H. Ouyang , Y. Luo , J. Xue , E. Wang , L. Zhang , Z. Zhou , Z. Liu , X. Li , S. Tan , Y. Chen , L. Nan , W. Cao , Z. Li , F. Chen , L. Zheng , Sci. Adv. 2024, 10, adi6799. 10.1126/sciadv.adi6799 PMC10776020 38181077

[13] M. Ali , M. J. Bathaei , E. Istif , S. N. H. Karimi , L. Beker , Adv. Healthcare Mater. 2023, 12, 2300318. 10.1002/adhm.202300318 PMC11469082 37235849

[14] X. Cui , L. Xu , Y. Shan , J. Li , J. Ji , E. Wang , B. Zhang , X. Wen , Y. Bai , D. Luo , C. Chen , Z. Li , Sci. Bull. 2024, 69, 1895. 10.1016/j.scib.2024.04.002 38637224

[15] H. Li , X. Zhang , L. Zhao , D. Jiang , L. Xu , Z. Liu , Y. Wu , K. Hu , M. R. Zhang , J. Wang , Y. Fan , Z. Li , Nano‐Micro Lett. 2020, 12, 50. 10.1007/s40820-020-0376-8 PMC7770853 34138256

[16] H. Feng , Y. Bai , L. Qiao , Z. Li , E. Wang , S. Chao , X. Qu , Y. Cao , Z. Liu , X. Han , R. Luo , Y. Shan , Z. Li , Small (Weinheim an der Bergstrasse, Germany) 2021, 17, 2101430. 10.1002/smll.202101430 34145752

[17] B. C. Heng , Y. Bai , X. Li , L. W. Lim , W. Li , Z. Ge , X. Zhang , X. Deng , Adv. Sci. (Weinh) 2023, 10, 2204502. 36453574 10.1002/advs.202204502 PMC9839869

[18] X. Zhang , T. Wang , Z. Zhang , H. Liu , L. Li , A. Wang , J. Ouyang , T. Xie , L. Zhang , J. Xue , Mater. Today 2023, 68, 117.

[19] Z. Liu , X. Wan , Z. L. Wang , L. Li , Adv. Mater. 2021, 33, 2007429. 10.1002/adma.202007429 34117803

[20] L. Chen , J. Yang , Z. Cai , Y. Huang , P. Xiao , J. Wang , F. Wang , W. Huang , W. Cui , N. Hu , Adv. Funct. Mater. 2024, 34, 2314079.

[21] B. Tandon , A. Magaz , R. Balint , J. J. Blaker , S. H. Cartmell , Adv. Drug Delivery Rev. 2018, 129, 148. 10.1016/j.addr.2017.12.012 29262296

[22] A. Proto , M. Penhaker , S. Conforto , M. Schmid , Trends Biotechnol. 2017, 35, 610. 28506573 10.1016/j.tibtech.2017.04.005

[23] R. Hinchet , S. W. Kim , ACS Nano 2015, 9, 7742. 26280752 10.1021/acsnano.5b04855

[24] G. T. Hwang , M. Byun , C. K. Jeong , K. J. Lee , Adv. Healthcare Mater. 2015, 4, 646. 10.1002/adhm.201400642 25476410

[25] J. Li , X. Wang , APL Mater. 2017, 5, 073801. 29270331 10.1063/1.4978936 PMC5734651

[26] M. Pan , C. Yuan , X. Liang , J. Zou , Y. Zhang , C. Bowen , iScience 2020, 23, 101682. 33163937 10.1016/j.isci.2020.101682 PMC7607424

[27] Y. Yu , H. Sun , H. Orbay , F. Chen , C. G. England , W. Cai , X. Wang , Nano Energy 2016, 27, 275. 28626624 10.1016/j.nanoen.2016.07.015 PMC5472384

[28] X. Chen , X. Li , J. Shao , N. An , H. Tian , C. Wang , T. Han , L. Wang , B. Lu , Small 2017, 13, 1604245. 10.1002/smll.201604245 28452402

[29] S. D. Mahapatra , P. C. Mohapatra , A. I. Aria , G. Christie , Y. K. Mishra , S. Hofmann , V. K. Thakur , Adv. Sci. (Weinh) 2021, 8, 2100864. 34254467 10.1002/advs.202100864 PMC8425885

[30] S. Mishra , L. Unnikrishnan , S. K. Nayak , S. Mohanty , Macromol. Mater. Eng. 2019, 304, 1800463.

[31] Y. Zhang , Y. Liu , Z. L. Wang , Adv. Mater. 2011, 23, 3004. 21560170 10.1002/adma.201100906

[32] J. Ji , C. Yang , Y. Shan , M. Sun , X. Cui , L. Xu , S. Liang , T. Li , Y. Fan , D. Luo , Advanced NanoBiomed Research 2023, 3, 2200088.

[33] J. H. Lee , J. Y. Park , E. B. Cho , T. Y. Kim , S. A. Han , T. H. Kim , Y. Liu , S. K. Kim , C. J. Roh , H. J. Yoon , H. Ryu , W. Seung , J. S. Lee , J. Lee , S. W. Kim , Adv. Mater. 2017, 29, 1606667. 10.1002/adma.201606667 28585262

[34] S. A. Han , T. H. Kim , S. K. Kim , K. H. Lee , H. J. Park , J. H. Lee , S. W. Kim , Adv. Mater. 2018, 30, 1800342. 10.1002/adma.201800342 29603416

[35] H. J. Shin , W. M. Choi , D. Choi , G. H. Han , S. M. Yoon , H. K. Park , S. W. Kim , Y. W. Jin , S. Y. Lee , J. M. Kim , J. Y. Choi , Y. H. Lee , J. Am. Chem. Soc. 2010, 132, 15603. 20945893 10.1021/ja105140e

[36] B. Kumar , K. Y. Lee , H. K. Park , S. J. Chae , Y. H. Lee , S. W. Kim , ACS Nano 2011, 5, 4197. 21495657 10.1021/nn200942s

[37] D. Wang , D. Zhang , P. Li , Z. Yang , Q. Mi , L. Yu , Nanomicro Lett 2021, 13, 57. 34138242 10.1007/s40820-020-00580-5 PMC8187675

[38] D. W. Jin , Y. J. Ko , C. W. Ahn , S. Hur , T. K. Lee , D. G. Jeong , M. Lee , C. Y. Kang , J. H. Jung , Small 2021, 17, 2007289. 10.1002/smll.202007289 33705597

[39] Z. Wen , Q. Shen , X. Sun , Nanomicro Lett 2017, 9, 45. 30393740 10.1007/s40820-017-0146-4 PMC6199050

[40] K. I. Park , M. Lee , Y. Liu , S. Moon , G. T. Hwang , G. Zhu , J. E. Kim , S. O. Kim , D. K. Kim , Z. L. Wang , K. J. Lee , Adv. Mater. 2012, 24, 2999. 22549998 10.1002/adma.201200105

[41] Y. Xue , T. Yang , Y. Zheng , K. Wang , E. Wang , H. Wang , L. Zhu , Z. Du , H. Wang , K. C. Chou , X. Hou , Adv. Sci. (Weinh) 2023, 10, 2300650. 37166066 10.1002/advs.202300650 PMC10288227

[42] J. H. Jung , M. Lee , J. I. Hong , Y. Ding , C. Y. Chen , L. J. Chou , Z. L. Wang , ACS Nano 2011, 5, 10041. 22098313 10.1021/nn2039033

[43] X. Wang , W. Z. Song , M. H. You , J. Zhang , M. Yu , Z. Fan , S. Ramakrishna , Y. Z. Long , ACS Nano 2018, 12, 8588. 30102853 10.1021/acsnano.8b04244

[44] S. Niu , Z. L. Wang , Nano Energy 2015, 14, 161.

[45] R. D. I. G. Dharmasena , K. D. G. I. Jayawardena , C. A. Mills , R. A. Dorey , S. R. P. Silva , Nano Energy 2018, 48, 391.

[46] F.‐R. Fan , Z.‐Q. Tian , Z. Lin Wang , Nano Energy 2012, 1, 328.

[47] Y. S. Zhou , S. Li , S. Niu , Z. L. Wang , Nano Res. 2016, 9, 3705.

[48] S. Niu , Y. Liu , S. Wang , L. Lin , Y. S. Zhou , Y. Hu , Z. L. Wang , Adv. Mater. 2013, 25, 6184. 24038597 10.1002/adma.201302808

[49] S. Niu , S. Wang , L. Lin , Y. Liu , Y. S. Zhou , Y. Hu , Z. L. Wang , Energy Environ. Sci. 2013, 6, 3576.

[50] H. Ouyang , Z. Liu , N. Li , B. Shi , Y. Zou , F. Xie , Y. Ma , Z. Li , H. Li , Q. Zheng , X. Qu , Y. Fan , Z. L. Wang , H. Zhang , Z. Li , Nat. Commun. 2019, 10, 1821. 31015519 10.1038/s41467-019-09851-1 PMC6478903

[51] Y. Su , G. Chen , C. Chen , Q. Gong , G. Xie , M. Yao , H. Tai , Y. Jiang , J. Chen , Adv. Mater. 2021, 33, 2101262. 10.1002/adma.202101262 34240473

[52] R. Qiu , X. Zhang , C. Song , K. Xu , H. Nong , Y. Li , X. Xing , K. Mequanint , Q. Liu , Q. Yuan , X. Sun , M. Xing , L. Wang , Nat. Commun. 2024, 15, 4133. 38755124 10.1038/s41467-024-48468-x PMC11099052

[53] G. Murillo , A. Blanquer , C. Vargas‐Estevez , L. Barrios , E. Ibanez , C. Nogues , J. Esteve , Adv. Mater. 2017, 29, 1605048. 10.1002/adma.201605048 28437016

[54] X. Meng , X. Xiao , S. Jeon , D. Kim , B. J. Park , Y. J. Kim , N. Rubab , S. Kim , S. W. Kim , Adv. Mater. 2023, 35, 2209054. 10.1002/adma.202209054 36573592

[55] I. M. Imani , B. Kim , X. Xiao , N. Rubab , B. J. Park , Y. J. Kim , P. Zhao , M. Kang , S. W. Kim , Adv. Sci. (Weinh) 2023, 10, 2204801. 36437039 10.1002/advs.202204801 PMC9875681

[56] C. Li , D. Liu , C. Xu , Z. Wang , S. Shu , Z. Sun , W. Tang , Z. L. Wang , Nat. Commun. 2021, 12, 2950. 34011979 10.1038/s41467-021-23207-8 PMC8136475

[57] M. T. Chorsi , E. J. Curry , H. T. Chorsi , R. Das , J. Baroody , P. K. Purohit , H. Ilies , T. D. Nguyen , Adv. Mater. 2019, 31, 1802084. 10.1002/adma.201802084 30294947

[58] R. Wang , J. Sui , X. Wang , ACS Nano 2022, 16, 17708. 36354375 10.1021/acsnano.2c08164 PMC10040090

[59] J. Li , J. Deng , S. Zhang , W. Chen , J. Zhao , Y. Liu , Adv. Sci. (Weinh) 2023, 10, 2305128. 37888844 10.1002/advs.202305128 PMC10754097

[60] Q. Xu , X. Gao , S. Zhao , Y. N. Liu , D. Zhang , K. Zhou , H. Khanbareh , W. Chen , Y. Zhang , C. Bowen , Adv. Mater. 2021, 33, 2008452. 34033180 10.1002/adma.202008452 PMC11469329

[61] A. E. Naclerio , P. R. Kidambi , Adv. Mater. 2023, 35, 2207374. 10.1002/adma.202207374 36329667

[62] N. Izyumskaya , D. O. Demchenko , S. Das , Ü. Özgür , V. Avrutin , H. Morkoç , Adv. Electron. Mater. 2017, 3, 1600485.

[63] Y. Lin , J. W. Connell , Nanoscale 2012, 4, 6908. 23023445 10.1039/c2nr32201c

[64] E. M. T. Fadaly , A. Dijkstra , J. R. Suckert , D. Ziss , M. A. J. van Tilburg , C. Mao , Y. Ren , V. T. van Lange , K. Korzun , S. Kolling , M. A. Verheijen , D. Busse , C. Rodl , J. Furthmuller , F. Bechstedt , J. Stangl , J. J. Finley , S. Botti , J. E. M. Haverkort , E. Bakkers , Nature 2020, 580, 205. 32269353 10.1038/s41586-020-2150-y

[65] J. Ben , X. Liu , C. Wang , Y. Zhang , Z. Shi , Y. Jia , S. Zhang , H. Zhang , W. Yu , D. Li , X. Sun , Adv. Mater. 2021, 33, 2006761. 10.1002/adma.202006761 34050555

[66] W. Li , J. Li , Nano Res. 2015, 8, 3796.

[67] M. T. Ong , E. J. Reed , ACS Nano 2012, 6, 1387. 22196055 10.1021/nn204198g

[68] T. Zhang , Y. Li , L. Li , X. Dong , J. Chen , X. Mu , C. Zhang , Z. Chen , W. Gong , T. Li , T. Zhang , S. Cong , Z. Zhao , Nano Energy 2022, 99, 107375.

[69] L. Dong , J. Lou , V. B. Shenoy , ACS Nano 2017, 11, 8242. 28700210 10.1021/acsnano.7b03313

[70] F. Xue , J. Zhang , W. Hu , W. T. Hsu , A. Han , S. F. Leung , J. K. Huang , Y. Wan , S. Liu , J. Zhang , J. H. He , W. H. Chang , Z. L. Wang , X. Zhang , L. J. Li , ACS Nano 2018, 12, 4976. 29694024 10.1021/acsnano.8b02152

[71] S. Bairagi , S. W. Ali , Int. J. Energy Res. 2020, 44, 5545.

[72] W. Deng , T. Yang , L. Jin , C. Yan , H. Huang , X. Chu , Z. Wang , D. Xiong , G. Tian , Y. Gao , H. Zhang , W. Yang , Nano Energy 2019, 55, 516.

[73] Y. Huan , X. Wang , J. Fang , L. Li , J. Eur. Ceram. Soc. 2014, 34, 1445.

[74] L. Su , L. Zou , C. C. Fong , W. L. Wong , F. Wei , K. Y. Wong , R. S. Wu , M. Yang , Biosens. Bioelectron. 2013, 46, 155. 23542085 10.1016/j.bios.2013.01.074

[75] M. Kitsara , A. Blanquer , G. Murillo , V. Humblot , B. De , S. Vieira , C. Nogues , E. Ibanez , J. Esteve , L. Barrios , Nanoscale 2019, 11, 8906. 31016299 10.1039/c8nr10384d

[76] S. Chen , X. Tong , Y. Huo , S. Liu , Y. Yin , M. L. Tan , K. Cai , W. Ji , Adv. Mater. 2024, 2406192. 10.1002/adma.202406192 39003609

[77] W. Deng , Y. Zhou , A. Libanori , G. Chen , W. Yang , J. Chen , Chem. Soc. Rev. 2022, 51, 3380. 35352069 10.1039/d1cs00858g

[78] Z. Liu , S. Zhang , Y. M. Jin , H. Ouyang , Y. Zou , X. X. Wang , L. X. Xie , Z. Li , Semicond. Sci. Technol. 2017, 32, 064004.

[79] Y. Mao , M. Shen , B. Liu , L. Xing , S. Chen , X. Xue , Sensors (Basel) 2019, 19, 3310. 31357659 10.3390/s19153310 PMC6696300

[80] J. Liu , D. Chen , P. Wang , G. Song , X. Zhang , Z. Li , Y. Wang , J. Wang , J. Yang , Talanta 2020, 215, 120890. 32312434 10.1016/j.talanta.2020.120890

[81] S. Sharma , R. Aguilera , J. Rao , J. K. Gimzewski , Sci. Rep. 2019, 9, 9282. 31243301 10.1038/s41598-019-45730-x PMC6594950

[82] Z. Li , T. Zhang , F. Fan , F. Gao , H. Ji , L. Yang , J. Phys. Chem. Lett. 2020, 11, 1228. 31990196 10.1021/acs.jpclett.9b03769

[83] J. Feng , Y. Fu , X. Liu , S. Tian , S. Lan , Y. Xiong , ACS Sustainable Chem. Eng. 2018, 6, 6032.

[84] A. H. Rajabi , M. Jaffe , T. L. Arinzeh , Acta Biomater. 2015, 24, 12. 26162587 10.1016/j.actbio.2015.07.010

[85] K. Kapat , Q. T. Shubhra , M. Zhou , S. Leeuwenburgh , Adv. Funct. Mater. 2020, 30, 1909045.

[86] L. E. Shlapakova , M. A. Surmeneva , A. L. Kholkin , R. A. Surmenev , Materials Today Bio 2024, 25, 100950. 10.1016/j.mtbio.2024.100950 PMC10840125 38318479

[87] L. Wu , H. Gao , Q. Han , W. Guan , S. Sun , T. Zheng , Y. Liu , X. Wang , R. Huang , G. Li , Biomater. Sci. 2023, 11, 7296. 37812084 10.1039/d3bm01111a

[88] Y. Ma , H. Wang , Q. Wang , X. Cao , H. Gao , Chem. Eng. J. 2023, 452, 139424.

[89] J. Zhang , Q. Wang , X. Tang , M. Chai , N. Liu , Z. Jiang , X. Li , P. Chen , Nano Energy 2024, 132, 110382.

[90] K. K. Das , B. Basu , P. Maiti , A. K. Dubey , Appl. Mater. Today 2024, 39, 102332.

[91] D. Khare , B. Basu , A. K. Dubey , Biomaterials 2020, 258, 120280. 32810650 10.1016/j.biomaterials.2020.120280

[92] M. Sekkarapatti Ramasamy , V. Krishnamoorthi Kaliannagounder , A. Rahaman , C. H. Park , C. S. Kim , B. Kim , ACS Biomater. Sci. Eng. 2022, 8, 3542. 35853623 10.1021/acsbiomaterials.2c00459

[93] Q. Xu , W. Dai , P. Li , Q. Li , Z. Gao , X. Wu , W. Liu , W. Wang , Nano Res. 2024, 17, 7461.

[94] H.‐F. Guo , Z.‐S. Li , S.‐W. Dong , W.‐J. Chen , L. Deng , Y.‐F. Wang , D.‐J. Ying , Colloids and Surfaces B, Biointerfaces 2012, 96, 29. 22503631 10.1016/j.colsurfb.2012.03.014

[95] Z. Zhu , X. Gou , L. Liu , T. Xia , J. Wang , Y. Zhang , C. Huang , W. Zhi , R. Wang , X. Li , Acta Biomater. 2023, 157, 566. 36481503 10.1016/j.actbio.2022.11.061

[96] P. J. Gouveia , S. Rosa , L. Ricotti , B. Abecasis , H. Almeida , L. Monteiro , J. Nunes , F. S. Carvalho , M. Serra , S. Luchkin , Biomaterials 2017, 139, 213. 28622605 10.1016/j.biomaterials.2017.05.048

[97] A. Cafarelli , P. Losi , A. R. Salgarella , M. C. Barsotti , I. B. Di Cioccio , I. Foffa , L. Vannozzi , P. Pingue , G. Soldani , L. Ricotti , J. Mech. Behav. Biomed. Mater. 2019, 97, 138. 31121432 10.1016/j.jmbbm.2019.05.017

[98] L. Dong , C. Jin , A. B. Closson , I. Trase , H. C. Richards , Z. Chen , J. X. Zhang , Nano Energy 2020, 76, 105076.

[99] X.‐X. Wang , G.‐F. Yu , J. Zhang , M. Yu , S. Ramakrishna , Y.‐Z. Long , Prog. Mater. Sci. 2021, 115, 100704.

[100] H. Zhong , J. Huang , J. Wu , J. Du , Nano Res. 2021, 15, 787.

[101] C. Burger , B. S. Hsiao , B. Chu , Annu. Rev. Mater. Res. 2006, 36, 333.

[102] N. Wang , X. X. Wang , K. Yan , W. Song , Z. Fan , M. Yu , Y. Z. Long , ACS Appl. Mater. Interfaces 2020, 12, 46205. 32933256 10.1021/acsami.0c13938

[103] C. Wang , J. Wang , L. Zeng , Z. Qiao , X. Liu , H. Liu , J. Zhang , J. Ding , Molecules 2019, 834, 24. 30813599 10.3390/molecules24050834 PMC6429487

[104] J. Xue , T. Wu , Y. Dai , Y. Xia , Chem. Rev. 2019, 119, 5298. 30916938 10.1021/acs.chemrev.8b00593 PMC6589095

[105] M. Sahu , S. Hajra , H.‐G. Kim , H.‐G. Rubahn , Y. K. Mishra , H. J. Kim , Nano Energy 2021, 88, 106255.

[106] J. Xue , J. Xie , W. Liu , Y. Xia , Acc. Chem. Res. 2017, 50, 1976. 28777535 10.1021/acs.accounts.7b00218 PMC6589094

[107] A. Haider , S. Haider , I. Kang , Arab J Chem 2018, 11, 1165.

[108] Y. Yi , J. Song , P. Zhou , Y. Shu , P. Liang , H. Liang , Y. Liu , X. Yuan , X. Shan , X. Wu , Carbohydr. Polym. 2024, 334, 122039. 38553236 10.1016/j.carbpol.2024.122039

[109] C. Jiang , C. Wu , X. Li , Y. Yao , L. Lan , F. Zhao , Z. Ye , Y. Ying , J. Ping , Nano Energy 2019, 59, 268.

[110] L. Lan , J. Xiong , D. Gao , Y. Li , J. Chen , J. Lv , J. Ping , Y. Ying , P. S. Lee , ACS Nano 2021, 15, 5307. 33687191 10.1021/acsnano.0c10817

[111] Z. Qin , Y. Yin , W. Zhang , C. Li , K. Pan , ACS Appl. Mater. Interfaces 2019, 11, 12452. 30860346 10.1021/acsami.8b21487

[112] M. Kim , C. Choi , J. P. Lee , J. Kim , C. Cha , Small 2022, 18, 2107316. 10.1002/smll.202107316 35306738

[113] W. Wei , M. Wildy , K. Xu , J. Schossig , X. Hu , D. C. Hyun , W. Chen , C. Zhang , P. Lu , Langmuir 2023, 39, 10881. 37390484 10.1021/acs.langmuir.3c01038 PMC10413944

[114] M. M. Abolhasani , M. Naebe , M. Hassanpour Amiri , K. Shirvanimoghaddam , S. Anwar , J. J. Michels , K. Asadi , Adv. Sci. (Weinh) 2020, 7, 2000517. 32670767 10.1002/advs.202000517 PMC7341085

[115] R. Liu , L. Hou , G. Yue , H. Li , J. Zhang , J. Liu , B. Miao , N. Wang , J. Bai , Z. Cui , Adv. Fiber Mater. 2022, 4, 604.

[116] B. Yu , H. Yu , T. Huang , H. Wang , M. Zhu , Nano Energy 2018, 48, 464.

[117] J. Zhang , S. Hu , Z. Shi , Y. Wang , Y. Lei , J. Han , Y. Xiong , J. Sun , L. Zheng , Q. Sun , Nano Energy 2021, 89, 106354.

[118] J. Mo , Y. Liu , Q. Fu , C. Cai , Y. Lu , W. Wu , Z. Zhao , H. Song , S. Wang , S. Nie , Nano Energy 2022, 93, 106842.

[119] Y. Yang , Q. Huang , G. F. Payne , R. Sun , X. Wang , Nanoscale 2019, 11, 725. 30565620 10.1039/c8nr07123c

[120] T. Park , N. Kim , D. Kim , S. W. Kim , Y. Oh , J. K. Yoo , J. You , M. K. Um , ACS Appl. Mater. Interfaces 2019, 11, 48239. 31766842 10.1021/acsami.9b17824

[121] R. J. Moon , A. Martini , J. Nairn , J. Simonsen , J. Youngblood , Chem. Soc. Rev. 2011, 40, 3941. 21566801 10.1039/c0cs00108b

[122] Z. Chen , Y. Hu , H. Zhuo , L. Liu , S. Jing , L. Zhong , X. Peng , R‐c. Sun , Chem. Mater. 2019, 31, 3301.

[123] S. Fan , W. Chang , C. Fei , Z. Zhang , B. Hou , Z. Shi , H. Wang , Y. Hui , Cellulose 2022, 29, 8919.

[124] D. Klemm , F. Kramer , S. Moritz , T. Lindstrom , M. Ankerfors , D. Gray , A. Dorris , Angew Chem Int Ed Engl 2011, 50, 5438. 21598362 10.1002/anie.201001273

[125] HP Abdul Khalil , Y. Davoudpour , M. N. Islam , A. Mustapha , K. Sudesh , R. Dungani , M. Jawaid , Carbohydr. Polym. 2014, 99, 649. 24274556 10.1016/j.carbpol.2013.08.069

[126] B. Liu , L. Liu , B. Deng , C. Huang , J. Zhu , L. Liang , X. He , Y. Wei , C. Qin , C. Liang , S. Liu , S. Yao , Int. J. Biol. Macromol. 2022, 222, 1400. 36195224 10.1016/j.ijbiomac.2022.09.270

[127] F. Hu , J. Zeng , Z. Cheng , X. Wang , B. Wang , Z. Zeng , K. Chen , Carbohydr. Polym. 2021, 254, 117474. 33357928 10.1016/j.carbpol.2020.117474

[128] D. C. Wang , H. Y. Yu , D. Qi , Y. Wu , L. Chen , Z. Li , J. Am. Chem. Soc. 2021, 143, 11620. 34286968 10.1021/jacs.1c04710

[129] L. Liu , Z. Niu , L. Zhang , W. Zhou , X. Chen , S. Xie , Adv. Mater. 2014, 26, 4855. 24838633 10.1002/adma.201401513

[130] G. Zhao , S. Gong , H. Wang , J. Ren , N. Wang , Y. Yang , G. Gao , S. Chen , L. Li , International Journal of Precision Engineering and Manufacturing‐Green Technology 2021, 8, 855.

[131] S. A. Graham , B. Dudem , A. R. Mule , H. Patnam , J. S. Yu , Nano Energy 2019, 61, 505.

[132] M. Naguib , V. N. Mochalin , M. W. Barsoum , Y. Gogotsi , Adv. Mater. 2014, 26, 992. 24357390 10.1002/adma.201304138

[133] A. Laschewsky , A. Rosenhahn , Langmuir 2019, 35, 1056. 30048142 10.1021/acs.langmuir.8b01789

[134] M. A. Stager , B. Adhikari , A. Raichart , M. D. Krebs , ACS Macro Lett. 2023, 12, 65. 36574625 10.1021/acsmacrolett.2c00713

[135] Z. Chen , Langmuir 2022, 38, 4483. 35380850 10.1021/acs.langmuir.2c00512

[136] A. Katchalsky , MIR. Polyampholytes , Journal of Polymer Science 1954, 13, 57.

[137] Q. Shao , S. Jiang , Adv. Mater. 2015, 27, 15. 25367090 10.1002/adma.201404059

[138] J. G. McDaniel , A. Yethiraj , J. Phys. Chem. Lett. 2018, 9, 4765. 30078326 10.1021/acs.jpclett.8b02120

[139] J. Cardoso , A. Huanosta , O. Manero , Macromolecules 1991, 24, 2890.

[140] C. J. Lee , H. Wu , Y. Hu , M. Young , H. Wang , D. Lynch , F. Xu , H. Cong , G. Cheng , ACS Appl. Mater. Interfaces 2018, 10, 5845. 29384644 10.1021/acsami.7b15934

[141] L. Zhang , Z. Cao , T. Bai , L. Carr , E.‐M. JR , C. Irvin , B. D. Ratner , S. Jiang , Nat. Biotechnol. 2013, 31, 553. 23666011 10.1038/nbt.2580

[142] Z. Pan , J. Dorogin , A. Lofts , G. Randhawa , F. Xu , R. Slick , M. Abraha , C. Tran , M. Lawlor , T. Hoare , Adv. Healthcare Mater. 2024, 13, 2304397. 10.1002/adhm.202304397 38684223

[143] Z. Xu , R. Han , N. Liu , F. Gao , X. Luo , Sensors and Actuators B, Chemical 2020, 319, 128253.

[144] X. Xie , J. C. Doloff , V. Yesilyurt , A. Sadraei , J. J. McGarrigle , M. Omami , O. Veiseh , S. Farah , D. Isa , S. Ghani , I. Joshi , A. Vegas , J. Li , W. Wang , A. Bader , H. H. Tam , J. Tao , H. J. Chen , B. Yang , K. A. Williamson , J. Oberholzer , R. Langer , D. G. Anderson , Nat. Biomed. Eng. 2018, 2, 894. 30931173 10.1038/s41551-018-0273-3 PMC6436621

[145] T. Xu , L. Zhang , B. Song , X. Bai , Z. Huang , X. Bu , T. Chen , H. Fu , P. Guo , J. Colloid Interface Sci. 2022, 620, 14. 35405562 10.1016/j.jcis.2022.03.125

[146] W. Hu , X. Wei , L. Zhu , D. Yin , A. Wei , X. Bi , T. Liu , G. Zhou , Y. Qiang , X. Sun , Nano Energy 2019, 57, 600.

[147] Y.‐H. Lai , Y.‐H. Chen , A. Pal , S.‐H. Chou , S.‐J. Chang , E.‐W. Huang , Z.‐H. Lin , S.‐Y. Chen , Nano Energy 2021, 90, 106545.

[148] R. Sarkar , A. Pal , A. Rakshit , B. Saha , J. Surfactants Deterg. 2021, 24, 709.

[149] R. Wang , L. Che , Q. Feng , K. Cai , ACS Appl. Mater. Interfaces 2022, 14, 12038. 35238538 10.1021/acsami.1c23017

[150] S.‐H. Jeong , Y. Lee , M.‐G. Lee , W. J. Song , J.‐U. Park , J.‐Y. Sun , Nano Energy 2021, 79, 105463.

[151] Y. Wu , J. Zou , K. Tang , Y. Xia , X. Wang , L. Song , J. Wang , K. Wang , Z. Wang , Burns & Trauma 2024, 12, tkae013. 38957661 10.1093/burnst/tkae013 PMC11218788

[152] V. K. Kaliannagounder , N. P. M. J. Raj , A. R. Unnithan , J. Park , S. S. Park , S.‐J. Kim , C. H. Park , C. S. Kim , A. R. K. Sasikala , Nano Energy 2021, 85.

[153] C. D. McCaig , M. Zhao , BioEssays , news and reviews in molecular, cellular and developmental biology 1997, 19, 819. 9297973 10.1002/bies.950190912

[154] J. Kwon , H. Cho , Commun. Biol. 2022, 5, 1229. 36369514 10.1038/s42003-022-04204-z PMC9652255

[155] Z. Zhou , D. Qian , M. Minary‐Jolandan , ACS Biomater. Sci. Eng. 2016, 2, 929. 33429502 10.1021/acsbiomaterials.6b00021

[156] A. C. Ahn , A. J. Grodzinsky , Medical engineering & physics 2009, 31, 733. 19286413 10.1016/j.medengphy.2009.02.006 PMC2771333

[157] L. Wang , Y. Pang , Y. Tang , X. Wang , D. Zhang , X. Zhang , Y. Yu , X. Yang , Q. Cai , Bioactive materials 2023, 25, 399. 37056250 10.1016/j.bioactmat.2022.11.004 PMC10087109

[158] A. K. Dubey , S. D. Gupta , B. Basu , Journal of biomedical materials research Part B, Applied biomaterials 2011, 98, 18. 21432997 10.1002/jbm.b.31827

[159] T. W. Sun , W. L. Yu , Y. J. Zhu , R. L. Yang , Y. Q. Shen , D. Y. Chen , Y. H. He , F. Chen , ACS Appl. Mater. Interfaces 2017, 9, 16435. 28481082 10.1021/acsami.7b03532

[160] G. Thrivikraman , P. S. Lee , R. Hess , V. Haenchen , B. Basu , D. Scharnweber , ACS Appl. Mater. Interfaces 2015, 7, 23015. 26418613 10.1021/acsami.5b06390

[161] K. Ravikumar , V. Kumaran , B. Basu , Biomaterials 2019, 209, 54. 31026611 10.1016/j.biomaterials.2019.04.010

[162] N. D. Ferson , A. M. Uhl , J. S. Andrew , IEEE transactions on ultrasonics, ferroelectrics, and frequency control 2021, 68, 229. 32866097 10.1109/TUFFC.2020.3020283

[163] X. Zhang , C. Zhang , Y. Lin , P. Hu , Y. Shen , K. Wang , S. Meng , Y. Chai , X. Dai , X. Liu , Y. Liu , X. Mo , C. Cao , S. Li , X. Deng , L. Chen , ACS Nano 2016, 10, 7279. 27389708 10.1021/acsnano.6b02247

[164] H. Chen , M. Liu , C. Jia , Z. Wang , IEEE transactions on ultrasonics, ferroelectrics, and frequency control 2009, 56, 2010. 19812004 10.1109/TUFFC.2009.1277

[165] V. Kovacova , J. I. Yang , L. Jacques , S. W. Ko , W. Zhu , S. Trolier‐McKinstry , Germany 2020, 26, 9356. 10.1002/chem.202000537 32274864

[166] N. More , G. Kapusetti , Medical hypotheses 2017, 108, 10. 29055380 10.1016/j.mehy.2017.07.021

[167] J. Jacob , N. More , K. Kalia , G. Kapusetti , Inflammation Regener. 2018, 38, 2. 10.1186/s41232-018-0059-8 PMC5828134 29497465

[168] A. Cafarelli , A. Marino , L. Vannozzi , J. Puigmartí‐Luis , S. Pané , G. Ciofani , L. Ricotti , ACS Nano 2021, 15, 11066. 34251189 10.1021/acsnano.1c03087 PMC8397402

[169] M. Ikram , M. A. P. Mahmud , Drug Delivery and Translational Research 2023, 13, 54. 35713781 10.1007/s13346-022-01184-9

[170] Q. Zheng , B. Shi , F. Fan , X. Wang , L. Yan , W. Yuan , S. Wang , H. Liu , Z. Li , Z. L. Wang , Advanced Materials (Deerfield Beach, Fla) 2014, 26, 5851. 25043590 10.1002/adma.201402064

[171] Q. Zheng , H. Zhang , B. Shi , X. Xue , Z. Liu , Y. Jin , Y. Ma , Y. Zou , X. Wang , Z. An , W. Tang , W. Zhang , F. Yang , Y. Liu , X. Lang , Z. Xu , Z. Li , Z. L. Wang , ACS Nano 2016, 10, 6510. 27253430 10.1021/acsnano.6b02693

[172] J. Tian , R. Shi , Z. Liu , H. Ouyang , M. Yu , C. Zhao , Y. Zou , D. Jiang , J. Zhang , Z. Li , Nano Energy 2019, 59, 705.

[173] L. Andrea , F. Adrien , G. Georg , R. Robert , B. Jan Niklas , T. Gregor , R. Anna Maria , V. J. B. J. J. Bjoern , Femoro‐Pedal Distraction in Staged Reconstructive Treatment of Tibial Aplasia 2020, 102, 1248. 10.1302/0301-620X.102B9.BJJ-2019-1484.R1 32862679

[174] C. Wang , H. Guo , P. Wang , J. Li , Y. Sun , D. Zhang Advanced materials (Deerfield Beach, Fla) 2023, 35, 2209895. 10.1002/adma.202209895 36738121

[175] N. Tang , Y. Zheng , M. Yuan , K. Jin , H. Haick , ACS Appl. Mater. Interfaces 2021, 13, 32106. 34223763 10.1021/acsami.1c06330

[176] M. Venkatesan , J. Chandrasekar , Y. C. Hsu , T. W. Sun , P. Y. Li , X. T. King , M. A. Chung , R. J. Chung , W. Y. Lee , Y. Zhou , J. H. Lin , C. C. Kuo , Adv. Sci. 2024, 11, 2404019. 10.1002/advs.202404019 PMC11425989 38981048

[177] Z. Li , D. He , B. Guo , Z. Wang , H. Yu , Y. Wang , S. Jin , M. Yu , L. Zhu , L. Chen , C. Ding , X. Wu , T. Wu , S. Gong , J. Mao , Y. Zhou , D. Luo , Y. Liu , Nat. Commun. 2023, 14, 6963. 37907455 10.1038/s41467-023-42598-4 PMC10618168

[178] X. Li , J. Wang , Y. Liu , T. Zhao , B. Luo , T. Liu , S. Zhang , M. Chi , C. Cai , Z. Wei , P. Zhang , S. Wang , S. Nie , Nano Lett. 2024, 24, 3273. 38427598 10.1021/acs.nanolett.4c00458

[179] L. Li , M. Yue , Q. Peng , X. Pu , Z. Li , Adv. Funct. Mater. 2023, 34, 2309594.

[180] S. Karunasagara , B. Bayarkhangai , H.‐W. Shim , H.‐J. Bae , H. Lee , A. Taghizadeh , Y. Ji , N. Mandakhbayar , H. S. Kim , J. Hyun , T.‐J. Kim , J.‐H. Lee , H.‐W. Kim , Biomaterials 2025, 314, 122854. 39405824 10.1016/j.biomaterials.2024.122854

[181] Z. Xia , H. Zhang , Q. Li , C. Yi , Z. Xing , Z. Qin , H. Zhao , J. Jing , C. Zhao , K. Cai , ACS Appl. Mater. Interfaces 2024, 16, 56730. 39394985 10.1021/acsami.4c11890

[182] D. Liu , X. Wang , C. Gao , Z. Zhang , Q. Wang , Y. Pei , H. Wang , Y. Tang , K. Li , Y. Yu , Q. Cai , X. Zhang , Adv. Mater. 2024, 36, 2409400. 10.1002/adma.202409400 39267457

[183] J. C. Silva , J. Meneses , F. F. F. Garrudo , S. R. Fernandes , N. Alves , F. C. Ferreira , P. Pascoal‐Faria , Sci. Rep. 2024, 14, 5458. 38443455 10.1038/s41598-024-55234-y PMC10915174

[184] A. Wang , X. Ma , Y. Yang , G. Shi , L. Han , X. Hu , R. Shi , J. Yan , Q. Guo , Y. Zhao , Nano Res. 2024, 17, 7376.

[185] M. Taale , F. Schütt , K. Zheng , Y. K. Mishra , A. R. Boccaccini , R. Adelung , C. Selhuber‐Unkel , ACS Appl. Mater. Interfaces 2018, 10, 43874. 30395704 10.1021/acsami.8b13631 PMC6302313

[186] O. Yue , X. Wang , M. Hou , M. Zheng , D. Hao , Z. Bai , X. Zou , B. Cui , C. Liu , X. Liu , Nano Energy 2023, 107, 108158.

[187] Y. Li , J. Chen , S. Liu , Z. Wang , S. Zhang , C. Mao , J. Wang , Matter 2024, 7, 1631.

[188] H. Yan , L. Li , Z. Wang , Y. Wang , M. Guo , X. Shi , J. M. Yeh , P. Zhang , ACS Biomater. Sci. Eng. 2020, 6, 634. 33463207 10.1021/acsbiomaterials.9b01601

[189] C. Boehler , F. Oberueber , M. Asplund , Journal of Controlled Release, Official Journal of the Controlled Release Society 2019, 304, 173. 31096016 10.1016/j.jconrel.2019.05.017

[190] T. Vinikoor , G. K. Dzidotor , T. T. Le , Y. Liu , H. M. Kan , S. Barui , M. T. Chorsi , E. J. Curry , E. Reinhardt , H. Wang , P. Singh , M. A. Merriman , E. D'Orio , J. Park , S. Xiao , J. H. Chapman , F. Lin , C. S. Truong , S. Prasadh , L. Chuba , S. Killoh , S. W. Lee , Q. Wu , R. M. Chidambaram , K. W. H. Lo , C. T. Laurencin , T. D. Nguyen , Nat. Commun. 2023, 14, 6257. 37802985 10.1038/s41467-023-41594-y PMC10558537

[191] A. Przekora , Int. J. Mol. Sci. 2019, 20, 435.30669519

[192] Z. Zhou , J. Zheng , X. Meng , F. Wang , Int. J. Mol. Sci. 2023, 24, 1836. 36768157 10.3390/ijms24031836 PMC9915254

[193] Y. Zhang , J. Tang , W. Fang , Q. Zhao , X. Lei , J. Zhang , J. Chen , Y. Li , Y. Zuo , Journal of Functional Biomaterials 2023, 185, 14. 10.3390/jfb14040185 PMC10146274 37103277

[194] M. R. Gomes , F. Castelo Ferreira , P. Sanjuan‐Alberte , Biomaterials Advances 2022, 137, 212808. 35929248 10.1016/j.bioadv.2022.212808

[195] S. M. Damaraju , Y. Shen , E. Elele , B. Khusid , A. Eshghinejad , J. Li , M. Jaffe , T. L. Arinzeh , Biomaterials 2017, 149, 51. 28992510 10.1016/j.biomaterials.2017.09.024

[196] Y. Li , X. Dai , Y. Bai , L. Y. , W. Y. , O. Liu , F. Yan , Z. Tang , X. Zhang , X. Deng , Int. J. Nanomed. 2017, 12, 4007. 10.2147/IJN.S135605 PMC5457183 28603415

[197] C. Alvarez‐Lorenzo , M. Zarur , A. Seijo‐Rabina , B. Blanco‐Fernandez , I. Rodríguez‐Moldes , A. Concheiro , Materials Today Bio 2023, 22, 100740. 10.1016/j.mtbio.2023.100740 PMC10374602 37521523

[198] B. Tandon , J. J. Blaker , S. H. Cartmell , Acta Biomater. 2018, 73, 1. 29673838 10.1016/j.actbio.2018.04.026

[199] J. Sun , W. Xie , Y. Wu , Z. Li , Y. Li , Adv. Sci. (Weinh) 2024, 2404190.

[200] S. Luo , C. Zhang , W. Xiong , Y. Song , Q. Wang , H. Zhang , S. Guo , S. Yang , H. Liu , Journal of Orthopaedic Translation 2024, 47, 191. 39040489 10.1016/j.jot.2024.06.009 PMC11261049

[201] Z. Liu , L. Xu , Q. Zheng , Y. Kang , B. Shi , D. Jiang , H. Li , X. Qu , Y. Fan , Z. L. Wang , Z. Li , ACS Nano 2020, 14, 8074. 32551540 10.1021/acsnano.0c00675

[202] P. Wu , L. Shen , H. F. Liu , X. H. Zou , J. Zhao , Y. Huang , Y. F. Zhu , Z. Y. Li , C. Xu , L. H. Luo , Z. Q. Luo , M. H. Wu , L. Cai , X. K. Li , Z. G. Wang , Military Medical Research 2023, 10, 35. 37525300 10.1186/s40779-023-00469-5 PMC10388535

[203] Z. Yang , Z. Yang , L. Ding , P. Zhang , C. Liu , D. Chen , F. Zhao , G. Wang , X. Chen , ACS Appl. Mater. Interfaces 2022, 14, 36395. 35925784 10.1021/acsami.2c08400

[204] A. García‐Fernández , B. Lozano‐Torres , J. F. Blandez , J. Monreal‐Trigo , J. Soto , J. E. Collazos‐Castro , M. Alcañiz , M. D. Marcos , F. Sancenón , R. Martínez‐Máñez , Journal of Controlled Release , Official Journal of the Controlled Release Society 2020, 323, 421. 32371265 10.1016/j.jconrel.2020.04.048

[205] H. Liu , Y. Shi , Y. Zhu , P. Wu , Z. Deng , Q. Dong , M. Wu , L. Cai , ACS Appl. Mater. Interfaces 2023, 15, 12273. 36890691 10.1021/acsami.2c19767

[206] A. Samadi , M. A. Salati , A. Safari , M. Jouyandeh , M. Barani , N. P. Singh Chauhan , E. G. Golab , P. Zarrintaj , S. Kar , F. Seidi , A. Hejna , M. R. Saeb , Journal of Biomaterials Science Polymer Edition 2022, 33, 1555. 35604896 10.1080/09205063.2022.2065409

[207] L. Wu , H. Gao , Q. Han , W. Guan , S. Sun , T. Zheng , Y. Liu , X. Wang , R. Huang , G. Li , Biomater. Sci. 2023, 11, 7296. 37812084 10.1039/d3bm01111a

[208] M. Bielfeldt , K. Budde‐Sagert , N. Weis , M. Buenning , S. Staehlke , J. Zimmermann , N. Arbeiter , S. Mobini , M. U. González , H. Rebl , A. Uhrmacher , U. van Rienen , B. Nebe , J. Biol. Eng. 2023, 17, 71. 37996914 10.1186/s13036-023-00393-1 PMC10668359

[209] S. Ghosh , W. Qiao , Z. Yang , S. Orrego , P. Neelakantan , Journal of Functional Biomaterials 2022, 8, 14. 10.3390/jfb14010008 PMC9867283 36662055

[210] C. Ning , Z. Zhou , G. Tan , Y. Zhu , C. Mao , Prog. Polym. Sci. 2018, 81, 144. 29983457 10.1016/j.progpolymsci.2018.01.001 PMC6029263

[211] H. Lee , S. Kim , S. Shin , J. Hyun , Carbohydr. Polym. 2021, 253, 117238. 33278994 10.1016/j.carbpol.2020.117238

[212] N. Bhaskar , M. C. Kachappilly , V. Bhushan , H. J. Pandya , B. Basu , J. Biomed. Mater. Res., Part A 2023, 111, 340. 10.1002/jbm.a.37472 36403282

[213] S. Dong , Y. Zhang , Y. Mei , Y. Zhang , Y. Hao , B. Liang , W. Dong , R. Zou , L. Niu , Frontiers in Bioengineering and Biotechnology 2022, 10, 921284. 35957647 10.3389/fbioe.2022.921284 PMC9358035

[214] C. Zhang , W. Wang , X. Hao , Y. Peng , Y. Zheng , J. Liu , Y. Kang , F. Zhao , Z. Luo , J. Guo , Adv. Funct. Mater. 2021, 31, 2007487.

[215] Un Dong , EJL Lim , J. K. Jang , S. Kim , Su Yeon Lee , T‐Ho Kim , Adv. Funct. Mater. 2024, 34, 2401717.

[216] Y. Wu , Y. X. Chen , J. Yan , D. Quinn , P. Dong , S. W. Sawyer , P. Soman , Acta Biomater. 2016, 33, 122. 26821341 10.1016/j.actbio.2016.01.036

